# Bridging the Synaptic Gap: *Neuroligins* and *Neurexin I* in *Apis mellifera*

**DOI:** 10.1371/journal.pone.0003542

**Published:** 2008-10-31

**Authors:** Sunita Biswas, Robyn J. Russell, Colin J. Jackson, Maria Vidovic, Olga Ganeshina, John G. Oakeshott, Charles Claudianos

**Affiliations:** 1 University of Queensland, Queensland Brain Institute, Brisbane, Queensland, Australia; 2 Australian National University, Research School of Biological Sciences, Canberra, Australian Capital Territory, Australia; 3 CSIRO Entomology, Black Mountain, Canberra, Australian Capital Territory, Australia; Wellcome Trust Sanger Institute, United Kingdom

## Abstract

Vertebrate studies show neuroligins and neurexins are binding partners in a trans-synaptic cell adhesion complex, implicated in human autism and mental retardation disorders. Here we report a genetic analysis of homologous proteins in the honey bee. As in humans, the honeybee has five large (31–246 kb, up to 12 exons each) *neuroligin* genes, three of which are tightly clustered. RNA analysis of the *neuroligin-3* gene reveals five alternatively spliced transcripts, generated through alternative use of exons encoding the cholinesterase-like domain. Whereas vertebrates have three *neurexins* the bee has just one gene named *neurexin I* (400 kb, 28 exons). However alternative isoforms of bee *neurexin I* are generated by differential use of 12 splice sites, mostly located in regions encoding LNS subdomains. Some of the splice variants of bee neurexin I resemble the vertebrate α*-* and β*-neurexins*, albeit in vertebrates these forms are generated by alternative promoters. Novel splicing variations in the 3′ region generate transcripts encoding alternative trans-membrane and PDZ domains. Another 3′ splicing variation predicts soluble neurexin I isoforms. *Neurexin I* and *neuroligin* expression was found in brain tissue, with expression present throughout development, and in most cases significantly up-regulated in adults. Transcripts of *neurexin I* and one *neuroligin* tested were abundant in mushroom bodies, a higher order processing centre in the bee brain. We show neuroligins and neurexins comprise a highly conserved molecular system with likely similar functional roles in insects as vertebrates, and with scope in the honeybee to generate substantial functional diversity through alternative splicing. Our study provides important prerequisite data for using the bee as a model for vertebrate synaptic development.

## Introduction

Vertebrate neuroligins and neurexins are trans-membrane cell adhesion molecules found predominantly on the post- and pre-synaptic membrane of synapses, respectively [1], [2]. Together they form an adhesion complex, which bridges the post- and pre-synaptic compartments of a synapse, thereby facilitating the development, specification and maintenance of a mature synapse.

*Neuroligin* genes have been identified in all animal genomes characterised but have been most thoroughly analysed in humans, mouse and rat [3], [4]. Three *neuroligin* genes are found in the rodents, and five in the human genome. Automated annotation of sequenced invertebrate genomes has identified *neuroligins* in the fruit fly (*Drosophila melanogaster*), mosquito (*Anopheles gambiae*), nematode worm (*Caenorhabditis elegans*) and honeybee (*Apis mellifera*) [5], [6]. Neuroligins are type I trans-membrane proteins comprised of a cleavable signal peptide, a large extracellular cholinesterase-like domain, EF-hand metal binding motifs, a short (O-linked) carbohydrate binding region linked to a single trans-membrane domain and a short cytosolic tail containing a PDZ (*P*ostsynaptic density 95/*D*iscs large/*Z*ona occludens 1) binding motif [7]. The extracellular cholinesterase-like domain of neuroligins participates in binding with neurexin. This domain possesses an α/β-hydrolase fold structure characteristic of the carboxyl-cholinesterase superfamily [8]–[10]. Although they lack the key active site residues required for catalytic competence as an esterase, the neuroligins are in fact the closest structural relative of the critical neurological enzyme acetylcholinesterase (AChE; [10]). Their amino acid sequence identity is low (∼30%) and the two proteins appear to have diverged before the divergence of the metazoa and porifera (C.Claudianos unpublished), but none-the-less some functional redundancy exists. Non-catalytic roles for AChE have been well established [11], [12], including a redundant neuritogenesis capacity shared with the neuroligins whereby both are able to affect neurexin expression [13], [14].

Alternative splicing of the vertebrate neuroligins arises from differential use of sites localised within the cholinesterase-like domain. A single site of alternative splicing (splice site A) has been found in human *neuroligins-1, -2* and *-3*, with an additional site (splice site B) also found in *neuroligin-1* [15]. However alternative splicing has not yet been reported for human *neuroligins -4X* and *-4Y* or any invertebrate neuroligins. Alternative splicing of the vertebrate neuroligins is critical to neuroligin-neurexin biology, in part determining whether an interaction occurs with either α-neurexin or β-neurexin [7], [16].

*Neurexin* genes have also been identified in all vertebrate and invertebrate genomes sequenced thus far. The well characterised mammalian systems all have three *neurexin* genes, each with both an upstream and downstream promoter [17]. The upstream promoter generates a larger protein called α-neurexin, whilst the downstream promoter gives rise to a smaller product called β-neurexin. The α- and β-neurexins are both single-pass trans-membrane proteins comprising a signal peptide, extracellular domain, trans-membrane domain, carbohydrate region and cytoplasmic tail [18]. The α-neurexins have a large extracellular domain made up of three repeats: each made up of two LNS (*L*aminin, *N*eurexin, *S*ex hormone-binding globulin) motifs flanking an EGF (*E*pidermal *G*rowth *F*actor) motif. The β-neurexins have a single LNS motif and a unique N terminal stretch. In addition to the use of alternative promoters, vertebrate *neurexin* transcripts gain diversity through alternative splicing within exons encoding their extracellular domains. Vertebrate α*-neurexins* possess five alternative splice sites, two of which are also found in β*-neurexins* [19], [20]. Similar to alternative splicing in the neuroligins, alternative splicing of the neurexins controls binding between the neuroligins and neurexins [7], [16]. Alternative splicing has not yet been reported for any invertebrate neurexins [20].

Vertebrate studies show that both neuroligins and neurexins participate in bi-directional protein-protein interactions at the synapse. Their N-terminal regions interact with one another in the extracellular synaptic compartment [21]; and binding of calcium to the neurexin is necessary for this interaction to occur [15], [52]. They also both interact with a number of intracellular PDZ motif-containing partners via their C-termini. The PDZ-motif partners behave as scaffolding molecules which in turn interact with transmitter receptors, ion channels and signalling proteins [22]–[27]. The first direct evidence implicating the neuroligin-neurexin complex in synapse formation came from studies which used cultured neurons to illustrate that neuroligins can promote the development of functional presynaptic terminals by binding with neurexin [28]. Further mammalian work then illustrated the capacity of neurexin to induce the assembly of postsynaptic proteins in cultured cells [29], [30]. This has since been demonstrated in *Drosophila,* by *in vivo* neurexin I over-expression [31]. Increased synapse formation was observed when verterbrate neuroligin was over-expressed in cultured neurons [29], [30], [32]–[35] and, conversely, reduced synapse density and changes in synapse activity were found by RNAi knock down of *neuroligin* expression [32]. Intriguingly, knock-out studies of *neuroligins* (single, double and triple knock-outs) or *neurexin* in mice show no changes to the density of synaptic contacts [36], [37]. However the *neuroligin* knockouts, similar to *Drosophila* neurexin I loss of function mutants [31], show reduced synaptic transmission and network activity in the brainstem. Arguably neuroligins do not play a role in the initial formation of synaptic contacts *in vivo*, but instead are involved in directing synapse maturation.

Several lines of evidence now suggest that the complex affects synapse specificity through differential effects on inhibitory versus excitatory synapse development, and thus on the excitatory/inhibitory (E/I) synapse ratio in the brain [29], [32], [35], [38] These findings include evidence for clustering with downstream signalling and scaffolding molecules, such as gephyrin, and show differences amongst neuroligin isoforms in their influence on excitatory and inhibitory synapse function. This complements research implicating human neuroligins and neurexins in neuro-developmental psychiatric disorders where an imbalance in E/I ratio is thought to occur. Numerous studies have localised mutations to *neuroligin 3* and *4* in families affected by autism, Aspergers syndrome and X-linked mental retardation [39]–[42]. The disease mutations in *neuroligin 3* and *4* lead to loss of neurexin binding, loss of synaptogenic capability and retention in the endoplasmic reticulum [43], [44]. Recent studies have also identified a high frequency of neurexin structural variants in families affected with autism and schizophrenia [45], [46].

The aim of the current study is to establish the honey bee as an invertebrate model for investigating the neurobiology of the neuroligin/neurexin complex. The bee is a neurologically sophisticated organism with well understood social biology-offering an avenue for effective learning and memory assays. In particular this study characterises the genetics of the neuroligins and neurexins in the bee. We find five *neuroligin* genes in the bee, as in humans, but only one *neurexin* gene, with a single promoter. However, functional diversity is achieved by much higher levels of alternative splicing in the exons encoding the extracellular domains of both *neuroligin* and *neurexin* genes in the bee. The bee genes are heavily but differentially expressed during development and concentrated in the mushroom bodies, which is the higher order processing centre of the bee brain.

## Results

### *Neuroligin* genes, gene structure and orthologs

We conducted *in silico* homology based searches of the honeybee genome [47] using predicted fruit fly (*Drosophila melanogaster*) and mosquito (*Anopheles gambiae*) *neuroligin* sequences [48]. The searches confirmed five Beebase Glean-3 annotations, GB18720, GB10066, GB18290, GB18836 and GB13939, as putative *neuroligin* sequences [49] (‘BeeBase’ http://racerx00.tamu.edu/). Specific nucleotide primers were then used to PCR amplify five *neuroligin* cDNAs from reverse-transcribed adult honeybee brain RNA. These are named *AmNLG1-5* according to their presumptive orthologies with other invertebrate *neuroligins* (Figure 1.1). Sequence analysis from the cloned amplicons showed that *AmNLG -1*, -*3*, -*4* and -*5* were full length cDNAs, each about approximately 2.5 kb in length. Despite strong sequence conservation between predicted orthologs and various attempts using different primer and PCR conditions, we were unable to verify the 5′ coding regions of *AmNLG2*. This may be due in part to significant secondary structure in the mRNA transcript as a consequence of the high GC rich content at the 5′-end of the *AmNLG2* gene. Verified coding sequence, including 1645 bp of the 3′ portion of *AmNLG2*, is given in Figure S1 of the Supplementary Data.

BLAST analyses of the complete honeybee genome identified the chromosomal locations of the five newly cloned *neuroligins*. Four of five *neuroligins* are located on chromosome 9, whereas *AmNLG2* is located on chromosome 1 (Table 1). Comparison between physical maps of *neuroligins* from the honeybee, fly and mosquito shows that *NLG -1,-3* and *-4* form a tightly linked cluster of genes, with the same order and orientation of orthologs in all three genomes [49]. Considering that dipteran and hymenopteran insects diverged over 300 million years ago, the conservation of this microsynteny may reflect a selective constraint.

**Table 1:** *Neuroligins* and *Neurexin I* in the Honeybee.

Gene	Chromosome	Size of gene	*Size of mature transcript	Exon Number
*Am NLG1*	9	55.8 kb	2430 bp	12
*Am NLG2*	1	45.9 kb	2550 bp	8
*Am NLG3*	9	246 kb	2424 bp	11
*Am NLG4*	9	215 kb	2433 bp	10
*Am NLG5*	9	31.2 kb	2553 bp	9
*AmNrxI_A*	5	391 kb	4872 bp	27
*AmNrxI_B*	5	394 kb	3633 bp	23

* Size of mature transcript refers to length of the open reading frame cDNA (exons only) from start to stop codon, without introns.

The phylogenetic relationships of the inferred amino acid sequences of the honeybee, fly, beetle and nematode neuroligin sequences show these as clear 1∶1 orthologs (Figure 1.1). Although there are no obvious orthologs between vertebrates and insects, all the invertebrate molecules including a single *C. elegans* (nematode worm) neuroligin share a common ancestry with the vertebrate neuroligins via a single basal ortholog, the sea urchin neuroligin (Figure 1.1). Interestingly, what would seem to be distinct vertebrate and invertebrate gene radiations separated by hundreds of millions of years of evolution result in an equivalent number of neuroligins (five) occurring in both humans and honeybees. Five putative *neuroligin* sequences are also found in the mosquito genome, orthologs of the five honeybee *neuroligins* [49]. *Drosophila* however, has only four *neuroligins* and lacks an ortholog of AmNLG5 (GB13939). The *Drosophila* genome is often missing members of gene families that are otherwise conserved between other invertebrate genomes [47].

**Figure 1:**
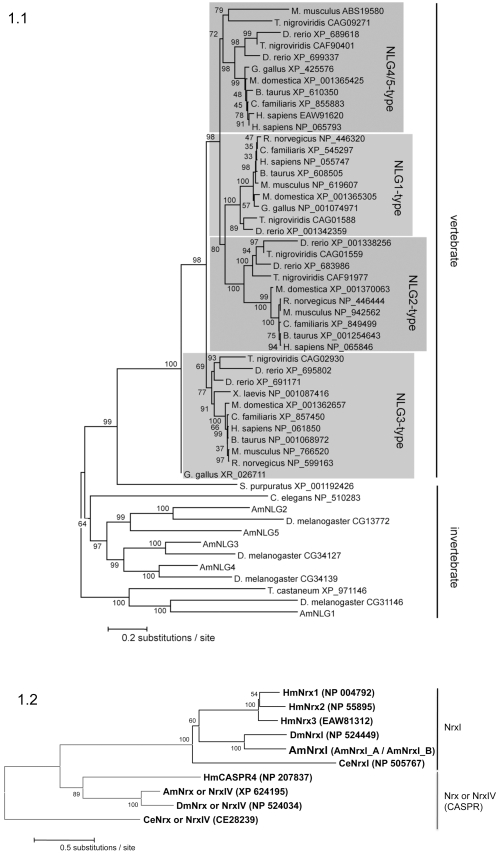
Neuroligin and Neurexin Phylogeny. (1.1) shows the phylogenetic relationship of 53 neuroligin proteins from the honeybee (AmNLG1-5) and *Drosophila* fly together with other neuroligins described by Bolliger et al [105]. All sequences are represented by taxon names showing species and NCBI accession numbers. The evolutionary history was inferred using the Neighbor-Joining method [106]. An optimal tree with percentage of replicate trees in which the associated taxa clustered together in the bootstrap test (1000 replicates) is shown next to the branches [107]. The tree is drawn to scale, with branch lengths in the same units as those of the evolutionary distances used to infer the phylogenetic tree. The evolutionary distances were computed using the JTT matrix based method [108] and are in the units of the number of amino acid substitutions per site. All positions containing alignment gaps and missing data were eliminated only in pairwise sequence comparisons (Pairwise deletion option). There were a total of 1884 positions in the final dataset. Phylogenetic analyses were conducted in MEGA3.1 [94]. Four radiations (shaded) represent relationship of vertebrate proteins NLG1-4 compared with invertebrate proteins. The phylogeny shows vertebrate and invertebrate neuroligin radiations arise from a single common ancestor found in the sea urchin (S. purpuratus XP_001192426), and displays a congruent topology with the Maximum-Likelihood tree reported by Bolliger et al. [105]. Similar analysis was performed for investigating neurexin phylogeny. (1.2) shows the evolutionary relationship of two ancestrally related clades of neurexin proteins from vertebrates and invertebrates; the neurological neurexins (NrxI) and neurexin IV (also known as neurexin). Notably, the invertebrate neurexin IV proteins form an orthologous group with CASPR. There were a total of 2125 positions in the final dataset. Also shown are the NCBI and wormbase accession numbers. Abbreviations Am: *Apis mellifera*, honeybee.

Genomic scaffolds (obtained from BeeBase) were analysed using SPIDEY (NCBI) to determine intron/exon arrangements of the honeybee *neuroligins* (Table 1). Canonical intron donor and acceptor splice site configurations (that typically contain GT/AG) were found to border the exon boundaries predicted from the homology searches and cDNA data. The numbers of exons and introns identified in the five genes were quite similar, around 8–12 each, with conservation of 8 intron/exon splice site junctions found between two or more of the honeybee neuroligins. All five genes have at least 10 fold more intron than exon sequence but the sizes of some introns varies widely between genes so that total gene length ranges from 31.2 kb (AmNLG5) to 246 kb (AmNLG3).

An alignment of predicted neuroligin proteins from the honeybee, fly, nematode and human indicates significant intron/exon splice site conservation among orthologs (the mosquito neuroligin sequences were not included in this analysis due to several incomplete gene predictions); for example, eight out of nine intron/exon splice sites in AmNLG3 are conserved in the *Drosophila* ortholog CG34127 (Figure S2). Splice site conservation amongst the honeybee neuroligin paralogs is lower than between orthologs in other species, but still discernable. The greater closeness between neuroligin orthologs than paralogs is also seen at the level of amino acid sequence identity; for example there is 27–48% amino acid identity between the honeybee paralogs compared with 59% identity between honeybee and *Drosophila* NLG3. Across the five honeybee neuroligins, there are 7–11 intron/exon splice junctions. Importantly, all five honeybee neuroligins possess the four intron/exon splice junctions found in the human neuroligins (Figure S2), and share an overall 24–32% amino acid identity with the five human sequences. These results suggest that a series of gene duplication events gave rise to the extant set of proteins and that their organisation has been substantively constrained over 800 million years of subsequent evolution.

### Neuroligin Proteins

Structural motifs characteristic of vertebrate neuroligins are generally present in the honeybee molecules; namely, a signal peptide, cholinesterase-like domain, EF-hand metal binding motifs and a trans-membrane domain (Figure S2). Critical arginine and valine C-terminal residues essential for the function of the PDZ (*P*ostsynaptic density 95/*D*iscs large/*Z*ona occludens 1) binding domain [22] are also present in all of the five honeybee neuroligins, suggesting that the intracellular protein-protein interactions may also be similar to their vertebrate counterparts.

Glycosylation in neuroligins has been shown to be physiologically important; specifically, N-linked glycosylation of vertebrate neuroligin 1 has been demonstrated to inhibit β-neurexin binding [7], [10]. Of the four sites of N-linked glycosylation that have been characterised in human neuroligins, namely N109, N303, N343 and N547 [10], only N547 is conserved in the honeybee and other invertebrate neuroligins. It may therefore be N547 which undergoes glycosylation in the invertebrate molecules as a method of regulating neurexin-neuroligin binding (Figure S2). O-linked glycosylation has also been shown to affect human neuroligin function. The two O-linked glycosylation sites (S683 and S686) are found in a proline-rich bottle brush-like structure that forms a ‘linker region’ between the esterase and trans-membrane domains [10], and is known to participate in neuroligin oligomerisation and mediate synaptogenic activity and neurexin binding [33]. The honeybee and *Drosophila* neuroligin 1 sequences possess equivalent O-linked glycosylation sites, and several additional serine and threonine residues are also present in this region (Figure S2). Thus O-linked glycosylation and oligomerisation of invertebrate neuroligins may follow the vertebrate model.

The recent publication of three crystal structures of human (*H. sapiens)* and mouse (*M. musculus*) neuroligin-neurexin complexes [50]–[52] allowed us to construct homology models of *Apis mellifera* NLG1 and NLG3 in order to analyse the sequence similarities and differences between the respective carboxyl/cholinesterase domains in greater detail. Although the mouse and human crystal structures are essentially identical in terms of sequence, the mouse structure was chosen as a template because of the marginally higher sequence conservation with the bee sequence (37% cf. 36% in respect to AmNLG3 for example). The alignments used to construct the models (Figures S6 and S7) illustrate that, with the exception of two 16 and 9 amino acid insertions in the AmNLG3 sequence, the proteins align closely.

Since cysteine residues play a critical role in defining the structure of the carboxyl/cholinesterase family members and they are highly conserved in vertebrate neuroligins [10], [53]–[55], potential disulfide bonds in the bee neuroligins were investigated through sequence alignment analysis and, specifically for AmNLG3, through the homology modelling analysis. The first two disulfide bridges appear to be conserved (C117-C153, C342, C353), while the third disulfide bridge (C512-C546) is absent, despite relatively high sequence identity in the nearby secondary structure elements. The loss of this inter-loop disulfide bridge suggests the conformation of this region in AmNLG-1 and -3 could be significantly different to that of human and mouse NLG1; interestingly, this disulfide bridge is located between the two helices (450–460;620–635) that constitute the dimerization domain in human and mouse neuroligins. A fourth disulfide bridge, found within a region of the vertebrate neuroligins that undergoes alternatively splicing [10] is also absent in the AmNLG3 sequence (Figure S7).

As shown in Figures 2.1a and b, sequence conservation is very strong in the interior of the protein, with the majority of the sequence variation occurring on its surface. In fact, there is 52% sequence identity between solvent-buried regions of the proteins compared to only 19% sequence identity in solvent-exposed regions. This result is consistent with the action of selective pressure to maintain the structural integrity of the α/β hydrolase fold, whereas there is apparently little selective pressure to retain similar sequences at the protein surface [56]. We are particularly interested in sequence conservation in functionally important areas of the neuroligin protein surface, such as the dimerization interface and the neuroligin/neurexin interface (discussed below). The recent elucidation of these interfaces in the mammalian proteins, initially revealed by low resolution X-ray scattering experiments [21] and followed by high resolution crystal structures [50]–[52], allowed us to identify the corresponding regions in our homology model.

**Figure 2:**
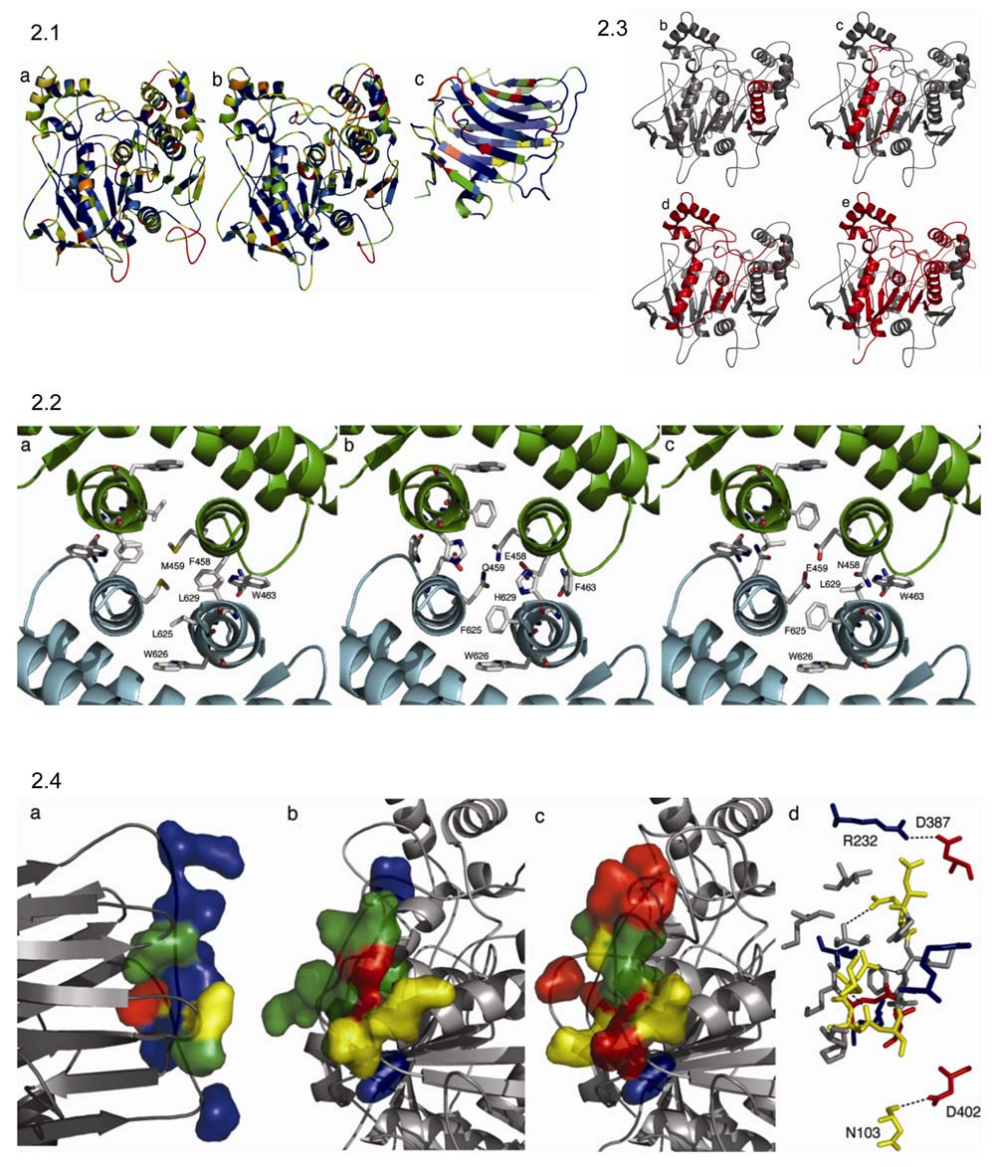
Structural Homology Modelling. (2.1) shows neuroligin and neurexin I homology models. The similarity between MmNLG1 and (a) AmNLG1 and (b) AmNLG3 is illustrated using colour, where sequence similarity in blue represents identical; green represents conservative; yellow represents semi-conservative and red represents dissimilar. Similarity to Mm β-Nrx1B with AmNrxI_A is illustrated using the same coloured coding. (2.2) shows the putative honeybee neuroligin dimer interfaces. The neuroligin dimer interface from (a) the crystal structure of mouse neuroligin is shown alongside the putative interfaces in (b) AmNLG1 and (c) AmNLG3. In both honeybee sequences the key residues of the ‘hydrophobic core’ are replaced by charged or polar residues. (2.3) illustrates the homology modelling of AmNLG3 aternative slice vriants. The four spliced variants (b–e) of AmNLG3 are shown. Full length AmNLG3 was modelled against mouse NLG1 [73]. Regions missing from the alternative transcripts are highlighted in red. (2.4) shows conservation of the neuroligin-neurexin interface in the honeybee. The respective surfaces of the neuroligin-neurexin interface are shown, based on the crystal structure of the complex from mouse. Sequence similarity is shown (blue, identical; green, conservative; yellow, semi-conservative; red, dissimilar) illustrating, (a) the strong conservation in AmNrxI-A, (b) the moderate conservation in AmNLG1, and (c) the lack of conservation in AmNLG3. (d) Illustrates a potential interaction between AmNrx1-A and AmNLG1, showing the conserved salt bridges (R232-D387), hydrogen bonds (N103-D402), and the potentially complementary hydrophobic and hydrophilic regions at the centre of the interface. Amino acids are coloured by type (blue, basic; red, acidic; yellow, polar; grey, non-polar).

Neuroligin dimerization in the human and mouse structures results from the withdrawal from solvent of an extensive hydrophobic region comprised from two α-helices from each monomer (α-12; 450–460, and α-18; 620–635). This symmetrical hydrophobic ‘core’ is principally formed by W626, L625 and L629 forming the top and bottom regions, with W463 and F459 forming the left and right sides, and a symmetrical M459-M459 interaction at the centre, which has been observed in the dimerization of other proteins [57]. The sequences of AmNLG1 and AmNLG3 are surprisingly different in this region (Figure 2.2). AmNLG1 retains the W626 and the hydrophobic character of L625(F) and W463(F), but the three central residues are either polar or charged (H629, E458, Q459). The conservation of hydrophobic residues at positions 625 and 626 would be necessary for correct intramolecular packing and folding of each monomer, but the increased charge of the residues at the other positions is likely to significantly affect inter-chain dimerization. Although it may be possible for these residues to arrange such that the dimer interface will be mediated by several hydrogen bonds, it is equally likely that these differences would prevent dimerization in an analogous fashion to the vertebrate structures. The corresponding regions of AmNLG3 also show differences that could compromise effective dimerisation. While the hydrophobic character at W626, L625 and W463 is again conserved, the central methionine is replaced by a glutamic acid, making it highly unlikely the two acidic side chains could pack closely together. However, given that the closely related acetylcholinesterases have been characterised in monomeric, dimeric and tetrameric forms [58], there is some precedent to suggest the possibility of differing molecular arrangements for the inverterbrate neuroligins. Furthermore, although it seems the dimerization contacts in human and mouse neuroligin are not conserved in AmNLG1 and 3, because of the other amino acid differences, including insertions and deletions, it is possible that an alternative dimerization mechanism may exist. The oligomeric state of the invertebrate enzymes and the molecular basis of their interactions thus remain to be experimentally verified.

Mutations to neuroligin genes have recently been linked to various neurological disorders. Mutations in human NL3 (R415C) and NL4 (D396Stop, G99S, K378R, V403M and R704C) have been identified in families affected by both mental retardation and autism [39]–[42]. With the exception of R704C, all of these mutations are associated with the carboxyl-cholinesterase domain of the neuroligins [42]. The D396 and R415C mutations are known to result in the retention of neuroligins in the endoplasmic reticulum, preventing expression of neuroligin at the postsynaptic membrane [43], [44]. Crystallographic analysis revealed that most of these mutations are in buried regions of the proteins, which supports the observation that they have detrimental effects on the structural integrity and folding of the protein [50]–[52]. Intriguingly, a number of these polymorphisms naturally occur within invertebrate neuroligins. For instance, the K278R and V403M changes are found in DmNLG1 and 3 (CG31146, CG34139) and the R415C change is found in AmNLG2 (Table 2; Figure S2). This suggests that the effects of these mutations could be more subtle than a loss of structural integrity and that they may be required for the specific functions of insect neuroligins. The wild type invertebrate neuroligins may therefore represent an interesting model that could be applied to the study of these autism-linked polymorphisms.

**Table 2:** Neuroligin (NLG) Disease-Associated Polymorphisms.

Gene	Disease Mutation K378R	Disease Mutation V403M	Disease Mutation R415C
Hm NLG1	K	V	R
Hm NLG2	K	V	R
Hm NLG3	K	V	R
Disease Hm NLG3* (Jamain et al. 2003)			C*
Hm NLG4X	K	V	R
Diesase Hm NLG4X* (Laumonnier et al. 2004)	R*	M*	
Hm NLG4Y	K	V	R
Am NLG1	L	A	R
Am NLG2	N	V	C
Am NLG3	A	V	K
Am NLG4	K	V	R
Am NLG5	G	I	R
Dm NLG1 (CG31146)	T	M*	R
Dm NLG2 (CG13772)	D	V	R
Dm NLG3 (CG34127)	S	V	R
Dm NLG4 (CG34139)	R*	V	R
Ce NLG	M	V	R

* Hm = human; Am = *Apis mellifera*; Dm = *Drosophila melangaster*; Ce = *Caenorhabditis elegans;* asterirsk = gene/citation/mutation found through familial disease data.

### *Neuroligin* alternative splicing

**Figure 3:**
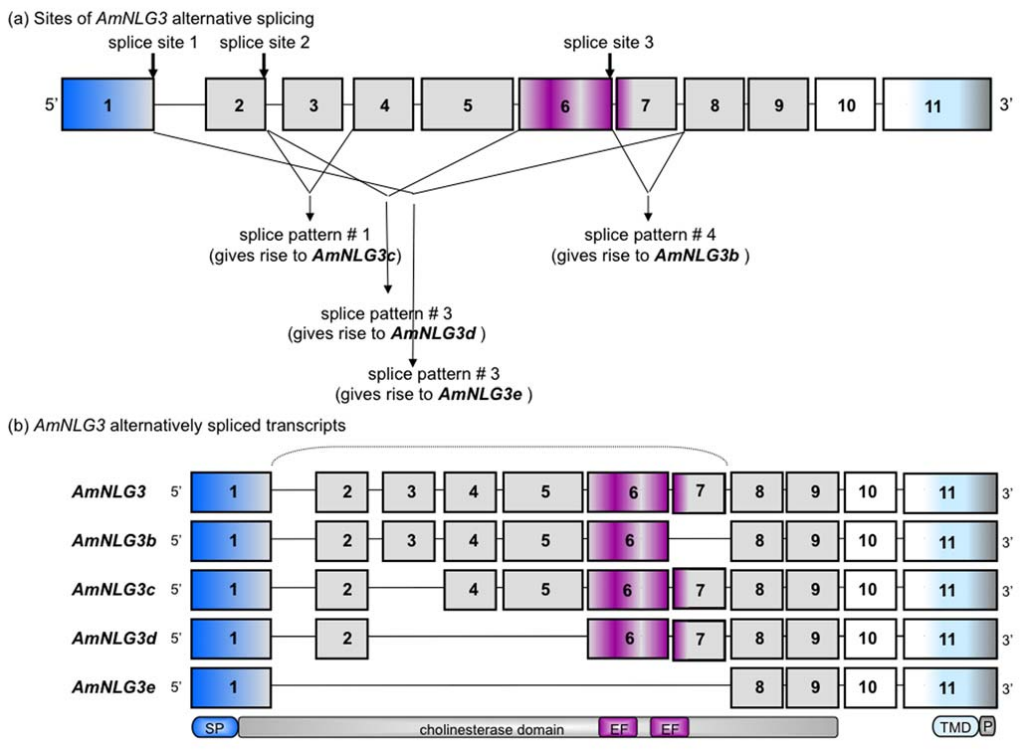
Honeybee *Neuroligin 3* Gene Arrangement and Intron/Exon Conservation. *AmNLG3* alternatively spliced transcripts were identified by RT-PCR from honeybee brain cDNA. Arrangement and intron/exon splice sites of *AmNLG3* splice variants were deciphered by both NCBI and Beebase BLAST tools against genomic DNA. Donor/acceptor splice sites were confirmed using NCBI SPIDEY. Sizes of exons, as well as intron gaps, are not drawn to scale. Exons are numbered from 5′ to 3′. (a) highlights the three sites of alternative splicing and resulting splice patterns. (b) illustrates the five alternatively spliced *AmNLG3* transcripts which correspond to the splicing patterns show in (a). The bracket highlights that splicing occurs within the cholinesterase domain. Signal peptide, cholinesterase, transmembrane domains and EF hand metal and PDZ binding motif are drawn below the encoding exons. Abbreviations SP: signal peptide; EF: EF hand metal binding motif; TMD: trans-membrane domain, P: PDZ binding domain.

Due to relatively high *AmNLG3* expression throughout development (discussed below), *AmNLG3* was chosen as a candidate to investigate alternative splicing. Candidate splice variants were amplified using RT-PCR and primers corresponding to the start and stop codons of the full-length gene (Table S1). Five alternatively spliced transcripts of *AmNLG3* were identified, including the full length transcript (Figure 3). These are denoted in decreasing order of transcript size *AmNLG3*, *AmNLG3b, AmNLG3c, AmNLG3d* and *AmNLG3e*. There are three splice sites which give rise to the five *AmNLG3* alternatively spliced transcripts. These coincide with the intron/exon splice junctions between exons 1–2, 2–3, and 6–7 (Figure S3). Comparison between the cDNA transcript sequence and corresponding genomic sequence revealed that all of the intron/exon boundaries of the five alternatively spliced transcripts are flanked by canonical AG/GT donor and acceptor splice sites [59]. In addition to amplification from expressed RNA, this relationship supports the conclusion that the amplicons represent truly expressed transcripts.

The region encoded between exons 2 and 7, in which all alternative splice sites of *AmNLG3* are located, encodes the adhesive carboxyl/cholinesterase domain (Figure 3.2, S3). All five AmNLG3 isoforms retain the signal peptide sequence, trans-membrane domain and intracellular cytosolic domain, suggesting all isoforms have the capacity to interact with the same intracellular postsynaptic proteins (Figure 3.2, S3). Importantly, the critical C-terminal ‘RV’ amino acids involved in PDZ binding are found in all five isoforms (Figure 3.2, S3).

There are two documented alternative splice sites in vertebrate *neuroligins*, the first of which is shared by *neuroligins 1*, *2* and *3* (site A), while the second (site B) is only found in *neuroligin 1* [55]. There is no alternative splicing attributed to humn *neuroligins 4X* and *4Y*. Comparison between the human neuroligins and the AmNLG3 variants shows that the first alternative splice site (A) of the human *neuroligins* is conserved in *AmNLG3* (Figure S3) and the second splice site (B) in human *NLG1* maps precisely to the intron/exon 3–4 splice junction in *AmNLG3*. Because there is a possibility that some RNA transcripts were not detected in the tissue or life stage from which the five cloned *AmNLG3* transcripts arose, it is conceivable that the second alternative splice in human *neuroligin 1* may also be a site of alternative splicing in the honeybee.

The most striking difference between the honeybee and vertebrate splice variants is the extent of the splicing: relatively short stretches of sequence(s) (9–19 amino acids) are spliced out of the vertebrate variants [55], whereas honeybee *neuroligin 3* shows whole exon splicing. The loss of exons 6 and/or exon 7 seen in *AmNLG3e*, for example, would result in the loss of the majority of the EF-hand metal binding motifs (Figure 3.2). The functional consequence of this loss is unclear given vertebrate studies have recently discovered that it is not neuroligin which binds calcium (via this motif), but neurexin [50]–[52]. The extent of the splicing is so extensive in AmNLG3e (9 of 11 β-strands of the central α-sheet are missing), that it is doubtful whether AmNLG3e, if folded, could assume an α/β hydrolase fold conformation. The splicing is also extensive in the other three splice variants. AmNLG3c and AmNLG3d lack one or two central β-strands (β-9 or β-9 and 10, respectively) in addition to several structurally important α-helices. AmNLG3b uniquely lacks β-9 in addition to two surrounding helices and the loop that in human and mouse neuroligins contacts β-neurexin 1 (Figure 2.3).

From a structural viewpoint, it is difficult to imagine how these splicing variants could correctly fold to give rise to soluble protein. There are, however, some observations that suggest these alterative transcripts may produce soluble protein. Firstly, all of the splice sites are within the carboxyl/cholinesterase domain. Secondly, the α/β-hydrolase fold that this domain adopts is notoriously flexible with respect to accommodating extensive alternative splicing, insertions and deletions, and still giving rise to viable protein [56]. Indeed, other instances of alternative splicing that produce viable protein resulting from the excision of entire domains have been reported, such as the vasopressin receptor [61]. Thus, in the absence of further empirical work, it remains possible that these isoforms are expressed and functionally relevant.

From another perspective, shorter alternatively spliced transcripts of other genes have been known to behave as a regulatory mechanism influencing the translation of their respective full length variants [60], [61]. *AmNLG3* splicing may therefore be behaving in a similar way. In fact, genome wide analysis of the honeybee genome has identified a micro RNA in association with *AmNLG2* [62]. Given micro RNAs are non-coding RNA molecules which can behave as potential regulators of gene expression at the post-transcriptional level, there is precedent to suggest that the various honeybee *neuroligins* (and thus specific variants) may be subject to differential degradation post-transcription.

Alternatively spliced transcripts of human *neuroligins 3* and *4X* were recently found to be unique to individuals affected by autism [63]. Intriguingly, the disease-associated splicing patterns give rise to transcripts that mimic alternatively spliced transcripts in the honeybee. Splice variants of human *neuroligin 4X* from autistic individuals are typically missing exon 2 of the open reading frame, which is an identical splicing pattern to that which gives rise to *AmNLG3c* (Figure S3). Likewise, splicing of *neuroligin 3* in autistic individuals results in the loss of exon 5 from the open reading frame, which aligns with exons 4 to 8 of *AmNLG3,* inclusively (Figures S2, S3). Although splicing of exon 8 has not been identified in the honeybee, exons 4, 5, 6 and 7 are alternatively spliced in varying combinations in *AmNLG3b*, *AmNLG3d* and *AmNLG3e*. Talebizadeh et al. [63] hypothesized that the disease-associated human isoforms could play a regulatory role by attenuating the function of the full-length isoform. The functional consequence of this may affect the balance between excitatory and inhibitory synapse development, similar to the regulatory mechanism of the vasopressin receptor protein [63], [64]. AmNLG3 alternate isoforms, therefore, may also play a role in regulating the function of the full-length molecule in the honeybee, albeit this would be part of the bee's normal biology.

### *Neurexin I* gene, gene structure and orthologs

Homology-based searches of the honeybee genome were performed using the putative *Drosophila neurexin* sequences CG7050 [65] and CG6827. Two honeybee *neurexin* sequences corresponding to Glean3 Beebase predictions GB18754 and GB14382 were identified. GB14382 proved to be the ortholog of the vertebrate *CASPR* gene, a non-neurological gene distantly related to the neurexins and not associated with neuroligin biology (see Figure 1.1). Therefore, GB14382 was not characterized further. However, the sequence of GB18754 showed a close match to *Drosophila Neurexin 1* (*DmNrx1*; CG7050) [65], except at the 3′ ends adjoining the stop codon. Specifically, the GB18754 sequence predicted a stop codon approximately 2.5 kb downstream of the stop codon found by homology based searching with CG7050.

To reconcile the alternative stop codons in the honeybee gene, two separate RT-PCR reactions were performed. Both used a common forward primer designed to the ATG start codon, but used different reverse primers designed to the two putative alternate stop codons (Figure 4.1, Table S1). Surprisingly, we amplified cDNAs for each of the alternative 3′-ends from brain RNA, confirming two types of transcripts are generated from the honeybee *neurexin 1* gene (*AmNrx1*). The 3′-end found through homology searching was further authenticated using 3′-RACE analysis. Nested primers were specifically designed to the sequence amplified cDNAs that encode C-terminal regions with adjacent untranslated sequences (900 bp). This included a polyadenylation site and a polyadenylated tail; thus confirming that the transcript arose from *bona fide* mature mRNA (Figure S9).

**Figure 4:**
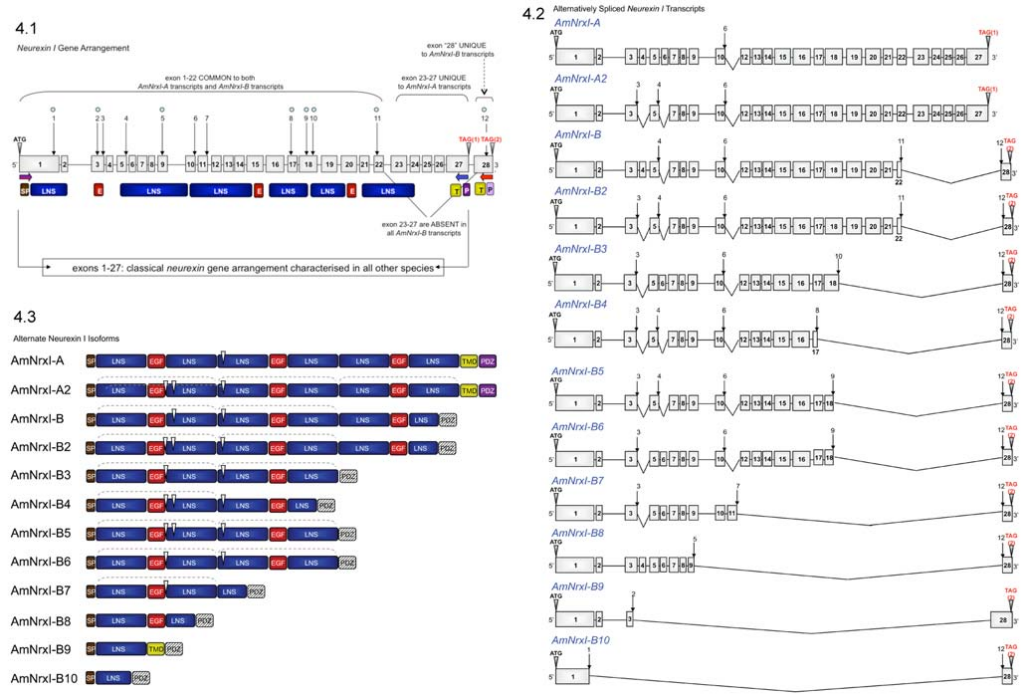
Gene Arrangement and Alternative Splicing of Honeybee *Neurexin I*. The gene arrangement and intron/exon splice sites of honeybee *neurexin I* and alternatively spliced transcripts were deciphered by both NCBI and Beebase BLAST tools against genomic DNA. Donor/acceptor splice sites were confirmed using NCBI SPIDEY. Multiple protein alignment analysis by ClustalW demonstrated the conservation of intron/exon splice sites (refer to Figures S4 and S5). Structural features were deciphered through PROSITE and SMART, and from Rissone et al. [109]; Jeleń et al. [71] and Sudhof et al. [110]. Exons are numbered from 5′ to 3. The size of exons, intron gaps and protein domains (and compared to one another) are not drawn to scale. Numbered arrows indicate sites of alternative splicing. (3.1) illustrates the full length *neurexin I* gene arrangement. The common start codon is highlighted by ATG. The two alternate stop codons are highlighted by TAG(1) and TAG(2). Sites of alternative splicing which occur within an exon are marked by a blue dot. Exons common to both the *AmNrxI-A* and *AmNrxI-B* transcripts are indicated above the gene schematic. Exons unique to *AmNrxI*–A transcript with a transmembrane domain are indicated above the gene schematic. The exon unique to the *AmNrxI-B* is labelled as ‘28’ and indicated as unique above. Horizontal primers below the gene schematic highlight the location of primers used for RT-PCR amplification. *AmNrxI-A* was amplified with the forward primer at exon 1 (ATG) and the reverse primer in blue at exon 27 (TAG1). *AmNrxI-B* was amplified with the same forward primer at exon 1 (ATG) and the reverse primer in red at exon 28 (TAG2). The protein domains which are encoded by particular exons are shown below the specific exons. The transmembrane domain and putative PDZ domain of AmNrxI-B are shown below exon 28. (3.2) illustrates the alternatively spliced *AmNrxI-A* and *AmNrxI-B* transcripts. (3.3) illustrates the alternate isoforms of AmNrxI-A and AmNrxI-B which arise from alternative splicing. White arrowheads indicate where splicing occurs. Brackets above highlight neurexin repeats (two complete LNS domains and an EGF motif). The putative PDZ domain of AmNrxI-B is hatched. Abbreviations- SP: signal peptide; E: EGF domain; T: transmembrane domain; P: PDZ binding motif. LNS domain: *L*aminin, *N*eurexin, *S*ex hormone-binding globulin.

*Neurexin I* is a ∼400 kb gene containing 28 exons located on chromosome 5 of the honeybee (Table 1). The two alternative stop codons are located in exons 27 and 28 (Figure 4.1, Figures S4, S5) and sequence analysis reveals exon 27 is absent from the honeybee *neurexin I* amplicon possessing exon 28. Thus each form of the *neurexin I* gene has a single stop codon. Exon 27 in the honeybee is orthologous to the 3′ terminal exon of *DmNrxI*. This arrangement is in fact typical of all other characterised invertebrate and vertebrate neurexins (Figure S4). Exon 28, on the other hand, represents a novel arrangement. This form is denoted *AmNrxI-B* while the conventional *neurexin I* variant using exon 27 is referred to as *AmNrxI-A*.

The mature *AmNrxI-A* and *AmNrxI-B* cDNA transcripts are 4.8 kb and 3.6 kb in size respectively (Table 1). They both span nearly 400 kb of genomic sequence, and contain 27 or 23 exons, respectively (Table 1). The first 22 exons are common to both, whereas exons 23 to 27 are unique to *AmNrxI-A* and exon 28 is only found in *AmNrxI-B* type transcripts (Figure 4.1). A similar number of exons (23 or 24) encode the vertebrate *neurexins*.

Translated full-length *AmNrxI-A* and *AmNrxI-B* were used together with the human, fly, mosquito and nematode proteins to determine a neurexin phylogeny. The translated honeybee GB14382 sequence (CASPR ortholog) was also included in this analysis (Figure 1.2). In contrast to the neuroligins, gene expansion within the ‘neurological’ *neurexin* family only occurs within the vertebrate lineage. The three vertebrate *neurexin* genes form an orthologous group with the one invertebrate *neurexin* gene (Figure 1.2). Like their vertebrate counterparts, the invertebrate *neurexin I* may thus also have neurobiological significance. The honeybee CASPR ortholog also has a *Drosophila* ortholog (which has been called *Drosophila neurexin IV*; [66] (Figure 1.2), and is expected to have non-neurological cell adhesion functions.

As expected, neurexin amino acid identity scores are higher among insects than between insect and vertebrates (55% and 30–33%, respectively). Surprisingly however, human and honeybee sequences are more alike in gene arrangement than honeybee is to *Drosophila*. Of 26 predicted AmNrxI intron/exon splice sites, 12 are shared with human neurexins 1–3, with another four intron/exon sites only slightly shifted between the species (25≤ residues). Ten out of 12 *Drosophila* sites are shared among the insect orthologs. The nematode *neurexin I* has 15 predicted intron/exon splice junctions, but surprisingly not one of these sites coincides with *Drosophila* neurexin I intron/exon splice junctions, whereas four of them are shared with the honeybee and human.

### Neurexin I Proteins

Analysis by PROSITE [67] and multiple protein alignment reveals obvious structural conservation between the characterised human neurexins and fly (*Drosophila)* and honeybee neurexin I. AmNrxI-A possesses a signal peptide, trans-membrane domain and C-terminal PDZ binding motif, as in the vertebrate neurexins (Figure 4.1 and Figure S4). As with their vertebrate orthologs (see introduction), AmNrx-A also contains three repeats, each consisting of two LNS (Laminin G-Neurexin-Sex hormone globulin) domains and a central epidermal growth factor (EGF) motif (i.e. three LNS-EGF-LNS repeats). AmNrxI-B is different to AmNrxI-A in two respects. Firstly, the sixth LNS domain (i.e. the second LNS in the third repeat) is incomplete in AmNrxI-B. LNS domains, specifically the sixth domain, have been shown to be involved in the invertebrate neurexin-neuroligin interaction [68], [69]. It is therefore reasonable to propose that AmNrxI-A and AmNrxI-B isoforms may have different neuroligin binding affinities. Secondly, AmNrxI-B lacks the trans-membrane domain and PDZ binding motif encoded by exon 27 in AmNrxI-A, suggesting that this isoform may be secreted or soluble (non-membrane bound). Soluble isoforms of neurexin, retaining three LNS-EGF-LNS repeats (Figure 4), have also been identified in humans, albeit these result from premature in-frame stop codons [70]. Due to the different genetic mechanisms by which soluble neurexin products arise in the bee and humans, the recurrence of this phenotype may be a case of convergent evolution. Interestingly, AmNrxI-B contains a short C-terminal VKTGVC sequence (exon 28) that encodes a conserved PDZ binding motif which includes diagnostic hydrophobic residues adjacent to the final cysteine [71], [72]. The respective functions of the AmNrxI isoforms will need to be determined experimentally; however, the primary sequence analysis presented here suggests that AmNrxI-B may still possess PDZ binding capacity. The physiological role of a soluble PDZ binding neurexin isoform is not obvious. One possibility is that this non-membrane form could interact with other, as yet unidentified, PDZ-type proteins.

### Neurexin *I* Alternative Splicing and Identification of a β-neurexin like Isoform

The extent of alternative splicing of honeybee *neurexin I* transcripts was investigated by conducting two series of RT-PCR reactions from honeybee cDNA, using primers corresponding to the predicted start and alternate stop codon regions of *AmNrxI-A* and *AmNrxI-B*. RT-PCR analysis of *AmNrxI-A* identified a spliced variant that we have called *AmNrxI-A2* (Figure 4, Figure S5). *AmNrxI-A2* results from the use of two alternative splice sites which remove segments of the gene corresponding to exons 4 and 6 of the full-length *AmNrxI-A* cDNA. Whereas AmNrxI-A possesses three LNS-EGF-LNS repeats, the putative AmNrxI-A2 isoform lacks part of the first EGF and second LNS domains. The first site of alternative splicing in *AmNrxI-A2* is equivalent to the first site of alternative splicing in the human *neurexins* (Figures S4, S5; [7]. In contrast, the second site of alternative splicing is unique to the honeybee. Of the four other alternative splice sites in the human neurexin, sites 2 and 4 coincide with intron/exon splice junctions in the honeybee gene.

RT-PCR analysis of *AmNrxI-B* identified ten alternatively spliced transcripts (denoted *AmNrxI-B-B10* in order of decreasing size; Figure 4, Figure S5). These ten transcripts arise through the differential use of twelve sites of alternative splicing. Eight of twelve splices sites occur within exons, while the remaining four occur at intron/exon boundaries. Interestingly, *AmNrxI-B7* contains an additional 72 bp exon (11) not found in any other *AmNrxI-A* or *AmNrxI-B* type transcripts. Thus, compared to this variant, *AmNrxI-A* in fact possesses three sites of alternative splicing, not two as would be inferred by comparison to *AmNrxI-A2*. It is possible that an *AmNrxI-A* transcript possessing exon 11 (plus all other exons in *AmNrxI-A*) exists within the honeybee transcriptome but was not detected in the tissue and developmental stages analysed here. Exon 11 encodes part of the third LNS domain (the first LNS of the second repeat).

Across the A and B forms of *neurexin I*, all of the alternative splice sites except splice sites 2 and 3 occur within LNS domains, suggesting alternative splicing will affect the neuroligin binding properties of these isoforms (Figure 4). In particular, the use of splice sites 1, 5, 7, 8 and 11 in *AmNrxI-B*, *B2*, *B7*, *B8* and *B10* give rise to truncated LNS domains. In addition to the truncated LNS domains, B family variants also differ in the number of LNS-EGF-LNS repeats. Full length human neurexin possesses three such repeats [18]. This arrangement is conserved in AmNrxI-A, -A2, -B and -B2, however AmNrxI-B3 to AmNrxI-B6 possess only two LNS-EGF-LNS repeats, and AmNrxI-B7 and -B8 possess just one (Figure 4). No such arrangements have been reported in neurexin literature before and raise interesting questions as to the function of these isoforms.

The AmNrxI-B9 and -B10 isoforms have a single LNS domain structure as per the vertebrate β-neurexins (Figure 4) [18]. Importantly, exon 28 is longer in *AmNrxI-B9* than in the other identified *AmNrxI-B* splice transcripts in which it is truncated (Figure 4). Because the full sequence of exon 28 encodes a trans-membrane domain immediately upstream of the putative PDZ binding domain (Figure 4, Figure S5), it is likely AmNrxI-B9 will be membrane bound and thus structurally similar to vertebrate β-neurexins. Furthermore, exon 28 of AmNrxI-B9 contains a serine rich region directly upstream of the trans-membrane domain (Figure S5); a similar serine-rich region, associated with O-glycosylation and a feature of cell surface receptors, is also found in the human neurexins where it confers trans-membrane orientation 17, 18.

### Conservation of the Neuroligin-Neurexin Interface in Honeybee

A homology model of AmNrxI-A was constructed using the recently crystallised neuroligin binding domain of mouse β-neurexin 1 (MmNrxβ1) as a template [50]–[52]. With the exception of two 3 amino acid insertions in the AmNrxI-A sequence, the domains aligned well and their overall sequence identity was relatively high (42%; Figure S8). Sequence identity was considerably higher in the protein core (58%) than the solvent exposed regions (25%), reflecting the selective pressure to maintain the same tertiary fold (Figure 2.1c). The exception to this trend was the neurexin-neuroligin interface, which unlike the rest of the protein surface, was highly conserved: of the thirteen residues that are buried to some extent upon neuroligin binding, six were identical (N103, P106, R232, I243, N238, S239), four were highly conserved (R109K, S132A, V154M, I236V) and three were semi-conservative differences (S107E, T108M, L135S). Importantly, the residues known to form salt bridges across this domain were conserved (R109K, R232). The calcium binding site residues (D137, V154, I234 and N236), essential for the neuroligin-neurexin interaction [51], are also located at this region and are also conserved, with the main chain carbonyl atoms of M145 and V236 being appropriately positioned with respect to the side chains of the analogous residues (D137 and N238) in neuroligin (Figures S6 and S7). Considering that there are million of years of divergent evolution between the bee and mouse, the similarity between these regions across the vertebrate and invertebrate neurexins suggests strong selective pressure to maintain this binding interface.

The complementary binding surface of the bee neuroligin 1 also shows moderate albeit incomplete conservation with vertebrate neuroligins. In AmNLG1, the level of conservation was less than that observed in AmNrxI, but was still significant: D387 and D402 are retained in AmNLG1 at each end of the interface (Figure S6) to potentially maintain a salt bridge and hydrogen bond with R232 and N103 of AmNrxI-A, respectively (Figure 2.4, Figure S8). There is less sequence identity in the central region of the interface in AmNLG1, although the hydrophobic character (G396P, F398F, F499A) and hydrogen bonding capability (Q395N, E397Q, N400R) of these residues are largely retained (Figure 2.4, Figure S8).

In contrast, the putative neuroligin-neurexin interface in AmNLG3 is remarkably divergent: of the eight residues that become buried on neurexin binding in MmNLG1, only D402 is conserved (Figure S7). Of particular note, both residues involved in salt bridges (D387P, N400R) are absent. In addition, there is a three amino acid insertion in the loop where the majority of the residues that form the interface are located (395–402) and a sixteen amino acid insertion immediately preceding it (Figure S7). Without over-interpretation of these models, we feel it is reasonable to propose the nature of the neuroligin-neurexin interaction will be conserved between vertebrate and invertebrates in the case of AmNrxI and AmNLG1. In contrast, it appears unlikely that AmNLG3 will interact with AmNrxI, at least in the same fashion as vertebrate NLG1 and β-Nrx 1. However, without resolution of the AmNLG3 and AmNrxI-A protein structures or other experimental means, this interaction cannot be ruled out. Furthermore, given differences in binding affinities have been documented between the human neuroligins and neurexin 1 [51], it is feasible to suggest that different binding affinities or interfaces exist between the bee neuroligins and neurexin I, thus shedding light on the differences seen by homology modelling of the AmNLG1-AmNrxI-A and AmNLG3-AmNrxI-A interactions.

### Expression Analyses

To elucidate expression patterns of honeybee *neurexin I* and the *neuroligins* in the developing brain, quantitative real time PCR (qRT-PCR) was performed from whole larvae RNA, pupal brain RNA and adult brain RNA of newly emerged, 3 day, 7 day and forager bees. For this work, primers were designed to regions of each gene that are common between the alternatively spliced transcripts (Table S1); thus, the profile for each gene should encompass all of the transcripts detected thus far.

**Figure 5:**
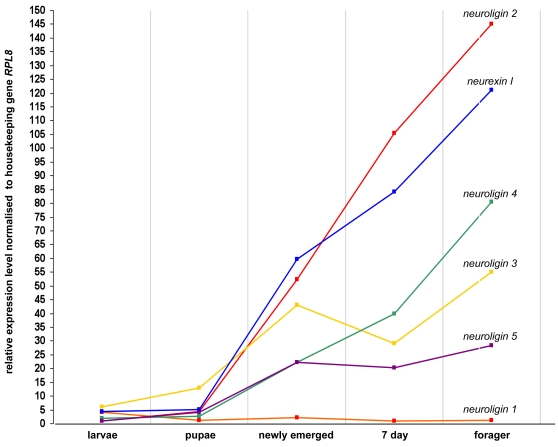
Developmental Expression Profiles of the *Neuroligins* and *Neurexin I* in Honeybee Brain. Honeybee *neuroligin* and *neurexin I* expression was assessed by quantitative real time PCR amplification. The ribosomal gene *RPL8* was used as the housekeeping gene. Methodology for data analysis and the presentation of results was taken from Collins et al [104]; where by expression levels were normalised by subtraction against the threshold cycle of the *RPL8*. Collins et al [104] found *RPL8* to be the best correlate with RNA concentration across varying developmental life stages and varying tissues of the honeybee. Expression levels were examined from whole larvae (5 day old); and brain tissue from pupae (stage P8 as outlined by Ganeshina et al [101]) 24 hour adult, 7 day adult and forager honeybees. Standards errors were negligible and less than +/−1.18 for all experimental results. The coloured lines illustrate the developmental expression profile of a single gene through development. Data points in columns illustrate the relative levels of *neurexin I* and *neuroligin* expression to one another at a particular stage of development. The developmental stage/gene with lowest expression relative to the control gene (neuroligin 1 at 7 days of age) was given an arbitrary expression level of 1. The data values are shown in Supplementary Data Table 3.

Expression of *neurexin I* and the *neuroligins* was found throughout development, from larvae to adult life stages (Figure 5, Table 3). Expression of *neurexin I* and *neuroligins 2, 3, 4* and *5* generally increases through development, with particularly pronounced up-regulation from pupal to adult stages. *Neuroligin 3* increases about 9 fold from early larvae to newly emerged adults (female worker bees), while *neurexin I*, *neuroligin 4* and *neuroligin 5* expression increases approximately 25–40 fold over this period. The expression of both *neuroligin 3* and *5* appears to drop slightly at one-week post adult emergence. *Neuroligin 2* shows the greatest change through development, with a 140-fold increase in expression through early developmental to adult stages. At the other extreme, *neuroligin 1* shows a consistent level of expression throughout development. It is also one of the most highly expressed *neuroligins* in the larval sample, although this could represent expression outside the central nervous system since whole larvae were analysed as opposed to only brain tissue at other developmental stages.

**Table 3:** Relative (Fold) Differences in Expression of Honeybee *Neuroligins* and *Neurexin I* through Development (Illustrated in Figure 5).

	larvae	pupae	newly emerged	7 day	forager
*neurexin I*	5.26	4.35	59.65	84.45	121.1
*neuroligin 1*	4.16	1.29	2.27	1	1.25
*neuroligin 2*	1.02	4.26	52.41	105.42	140.07
*neuroligin 3*	6.25	12.91	42.96	38.59	54.95
*neuroligin 4*	2.04	2.69	22.34	39.95	79.99
*neuroligin 5*	1.1	4.06	22.16	20.25	28.34

* numbers indicate relative fold difference in expression level of genes compared to one another, and relative fold difference in expression level of a single gene through development–all values normalised to *RPL8*. Raw data shown in Supplementary: Table A3, A3i and A3ii.

**Figure 6:**
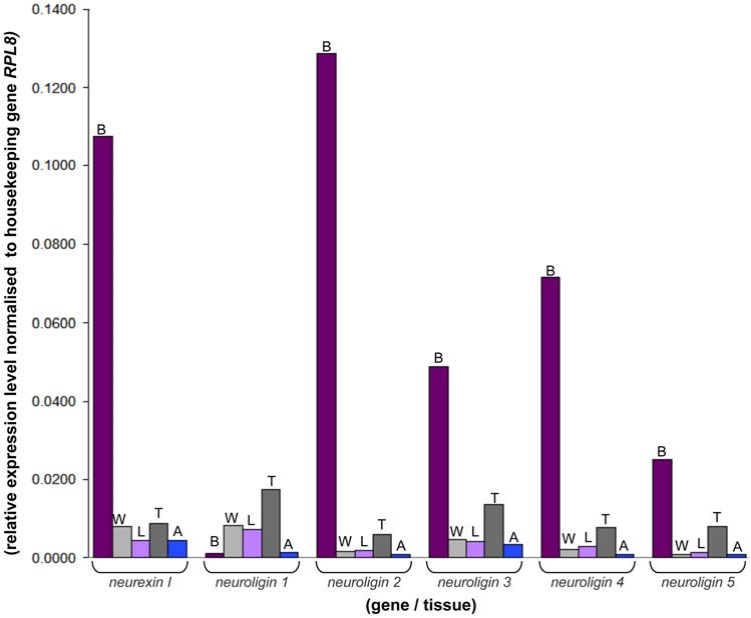
Spatial Expression of *Neuroligins* and *Neurexin I* in the Adult Honeybee. The methodologies behind attaining these results are as described in the Figure 5 legend. Expression levels are shown relative to the house keeping gene *RPL8,* given an arbitrary value of 1. Expression levels were examined from the tissue of ten adults at twenty-one days of age. Standards errors were negligible and less than +/−1.22 for all experimental results. Level of gene expression in the brain expression shown in the dark purple columns marked B, wings in the light grey columns marked W, legs in the light purple columns marked L, thorax in the dark grey columns marked T and abdomen in the blue columns marked A. Raw data from the qRT-PCR experiments in Supplementary Data Table 4.

qRT-PCR experiments show that all of the honeybee *neuroligin* genes and *neurexin I* are also expressed outside of the adult brain; in the thorax, legs, abdomen and wings (Figure 6, Table 4). Expression levels (relative to the housekeeping gene *Ribosomal Protein L8* (*RPL8*)) are still significantly higher in the brain compared to other tissues for *neuroligins 2–5* and *neurexin I* but *neuroligin 1* expression is much greater in tissues outside of the brain. This suggests a putative role for *neuroligin 1* in the peripheral nervous system in addition to the central nervous system, potentially with importance at neuro-muscular junctions. Furthermore, significant expression of *neuroligin 1* in the wings and legs (Table 4) suggests a putative role in nerve endings responsive to sensory input. Interestingly, human *neuroligin 4X* displays a similar expression profile to honeybee *neuroligin 1*, with low levels of brain expression and higher expression levels outside of the brain [4], [74]. Human *neuroligin 4X*, however, has been established as a critical molecule required for proper neuro-connectivity [39], [41], [42], illustrating that the relative distribution or expression level of a gene is not necessarily informative of its functional priorities. Thus the low levels of *neuroligin 1* expression in the honeybee brain may not necessarily suggest a trivial role. On another note, although *neuroligins* are expressed outside of the central nervous system in all characterised species, *neurexin* expression is strictly restricted to the human and rodent brain, suggesting that honeybee *neurexin I* has greater functional diversity than that seen in vertebrates.

**Table 4:** Relative (Fold) Differences in Expression of Honeybee *Neuroligin* and *Neurexin I* Spatial Expression in the Adult (Illustrated in Figure 6).

Gene	Tissue	Expression Level Relative to *RPL8*	Expression Level Relative to Brain Expression
*RPL8*	(housekeeping)	1.0000	
*NrxI*	Brain	0.1073	1.00
	Wing	0.0080	13.45 fold LESS
	Leg	0.0043	24.85 fold LESS
	Thorax	0.0089	12.13 fold LESS
	Abdomen	0.0043	24.85 fold LESS
*NLG1*	Brain	0.0011	1.00
	Wing	0.0082	7.390 fold MORE
	Leg	0.0073	6.620 fold MORE
	Thorax	0.0176	15.83 fold MORE
	Abdomen	0.0013	1.140 fold MORE
*NLG2*	Brain	0.1285	1.00
	Wing	0.0014	88.650 fold LESS
	Leg	0.0019	67.420 fold LESS
	Thorax	0.0059	21.710 fold LESS
	Abdomen	0.0008	161.46 fold LESS
*NLG3*	Brain	0.0487	1.00
	Wing	0.0046	10.48 fold LESS
	Leg	0.0040	12.13 fold LESS
	Thorax	0.0137	3.560 fold LESS
	Abdomen	0.0035	13.91 fold LESS
*NLG4*	Brain	0.0713	1.00
	Wing	0.0022	31.78 fold LESS
	Leg	0.0028	25.46 fold LESS
	Thorax	0.0078	9.090 fold LESS
	Abdomen	0.0008	86.52 fold LESS
*NLG5*	Brain	0.0251	1.00
	Wing	0.0009	26.63 fold LESS
	Leg	0.0014	18.00 fold LESS
	Thorax	0.0080	3.150 fold LESS
	Abdomen	0.0009	27.76 fold LESS

*In situ* hybridisation was used to investigate the distribution of *neurexin I* (*AmNrxI-A* and *AmNrxI-B*) and *neuroligin 3* (*AmNLG3*) within the honeybee brain. In order to detect the expression of all possible RNA transcripts, probes were again designed to regions that are common to all alternatively spliced transcripts. Specifically, the probes used to investigate *AmNrxI-A* and *AmNrxI-B* expression were designed to the common 5′ region of the gene. The probes used to investigate *AmNLG3* expression were not designed to the cholinesterase region where splicing is localised, but instead to the first and last exons of the gene which are common to all alternatively spliced transcripts.

*AmNLG3* is predominantly found in the mushroom body of the adult brain, with some expression also in the cell bodies of the optic lobes, antennal lobes and central body (Figure 7.1). A broadly similar distribution pattern is seen for *AmNrxI* whereby the transcripts are predominantly localised to the mushroom body of the adult brain (Figure 7.2). However, expression of *AmNrxI* is more dispersed throughout the pupal brain than in the adult, suggesting *AmNrxI* may have a general role in early brain development and a more specific role in the adult brain.

**Figure 7:**
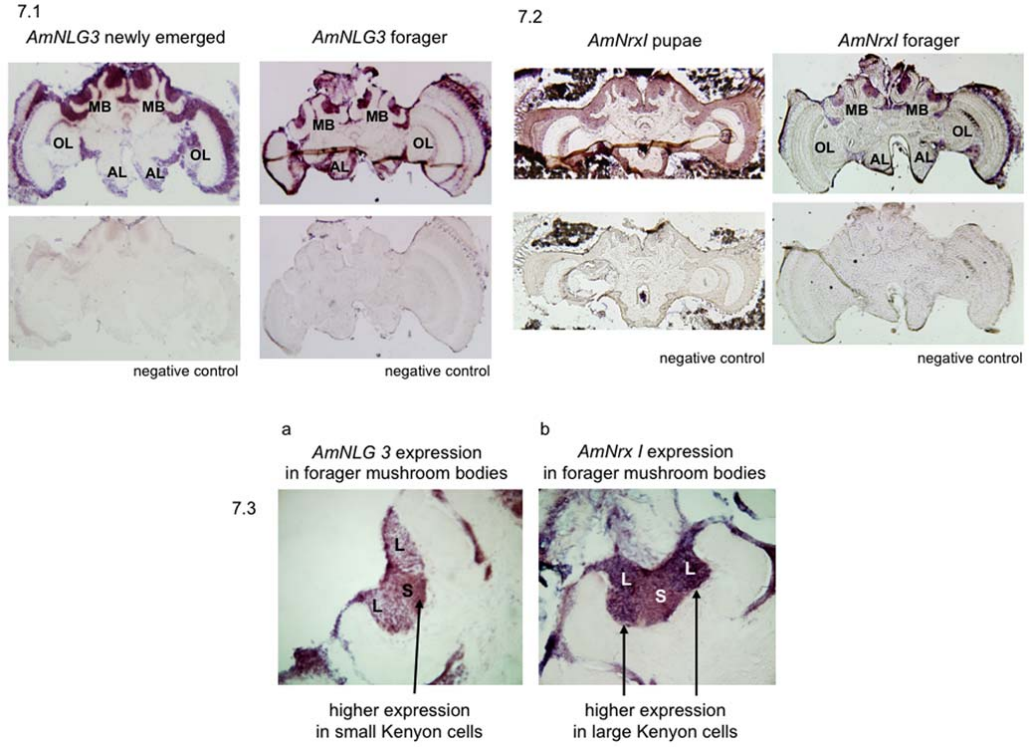
*Neuroligin* and *Neurexin I* Brain Expression. To identify RNA transcript distribution, *in situ* hybridisation experiments were performed on 20 µm honeybee brain sections using gene specific digoxygenein labelled probes: (7.1) illustrates expression of *AmNLG3* in the adult brain of newly emerged (left) and forager (right) bees. Top images illustrate results of anti-sense probe staining. Bottom images illustrate the results of the negative control experiments using a sense probe. (7.2) Expression of *AmNrxI*/*AmNrxI_28* in both a P8 stage (outlined by Ganeshina et al. [101]) pupae (left) and adult forager (right) honeybee brain. Top images illustrate the results of anti-sense probe staining. Bottom images illustrate results of the negative control experiments using a sense probe. (7.3) shows contrasting *AmNLG3* and *AmNrxI* expression in the adult mushroom body. (a) *AmNLG3* expression higher in the small Kenyon cells. (b) *AmNrxI* expression higher in the large Kenyon cells. Abbreviations- MB: mushroom body; OL: optic lobe; AL: antennal lobe; L: large Kenyon cells; S: small Kenyon cells.

The mushroom body in insects is considered the centre of higher order processing and a functional analogue of the hippocampus in vertebrates [75], [76]. The optic and antennal lobes relay visual and olfactory stimuli to the mushroom bodies [77], [78], thus the expression of *AmNLG3* and *AmNrxI* in these tissues is consistent with a role for these products in sensory signalling and cognitive processing. Changes in *neuroligin* and *neurexin I* gene expression in association with training bees under memory and learning tasks have been observed (S.Biswas unpublished), further strengthening the significance of this observed expression. A role for neurexin I in synaptogenesis and cognition has also been demonstrated in *Drosophila,* where neurexin I null mutants were found to exhibit decreased synapse number and associative learning defects [65].

A closer examination of the *in situ* hybridisation images revealed a difference between the expression patterns of *neuroligin 3* and *neurexin I* in the mushroom bodies. The mushroom body is made up of four encircling ‘cup like’ structures called calyces, each filled with intrinsic neurons called Kenyon cells [78]–[80]. The Kenyon cells are subdivided into two morphologically distinct types, the large-type and small-type Kenyon cells [78], [81]. Interestingly, expression of *neuroligin 3* was higher in the small-type central Kenyon cells than in the large-type peripheral Kenyon cells (Figure 7.3a). The reverse was seen for *neurexin I*, which was more highly expressed in the large-type than small-type Kenyon cells (Figure 7.3b). This difference is intriguing given the trans-synaptic partnership between *neuroligins* and *neurexins* [82]–[84] would predict a parallel expression pattern. On the other hand, differences in RNA localisation in the Kenyon cell bodies do not necessarily reflect the distribution of synaptic proteins that can be distantly localised on axonal projections in the neuropil [77], [78].

**Figure 8:**
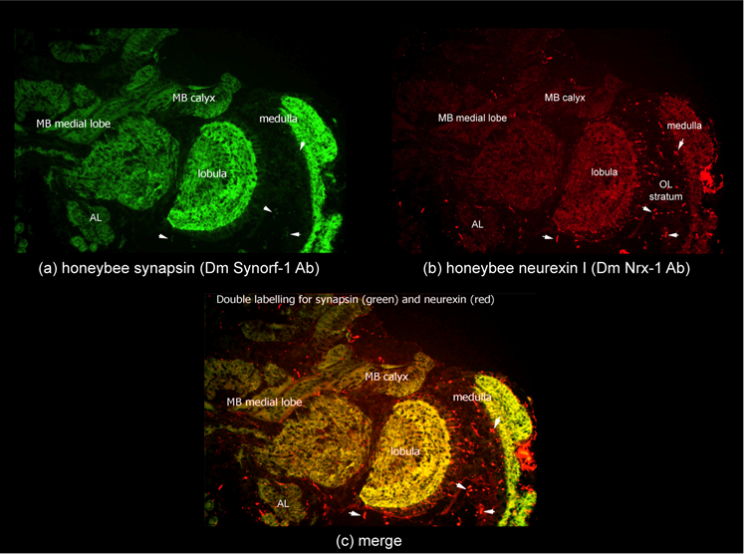
Honeybee *Neurexin I* Protein Expression in the Brain. Immuno-staining of forager brain sections (a) for synapsin using SynOrf-1 antibody and incubation with Alexa-488-conjugated anti-mouse antibody allowing green colouration to highlight protein expression; (b) for honeybee neurexin-I using DmNrx-1 antibody and incubation with Alexa-546-conjugated anti-mouse antibody allowing red colouration to highlight protein expression; (c) merge shows neurexin-I and synapsin co-localise in mushroom body neuropil (MB medial lobe and MB calyx), optic lobe neuropil (medulla and lobulla) and antennal lobes. Small puncta of neurexin I expression, distinct to synapsin, are highlighted by small arrow heads within the optic lobe (OL) stratum.

An immunohistochemical analysis was therefore conducted to assess the localisation of the honeybee neurexin I protein in the bee brain. For this we used a *Drosophila* neurexin I polyclonal antibody provided by Professor Wei Zie (Department of Genetics and Developmental Biology, Genetics Research Center, Southeast University Medical School, China; [65]) which cross reacts with honeybee neurexin I (Figure 8). As with the Zeng et al. [65] study, we used the monoclonal antibody to *Drosophila* synapsin (SynOrf-1), known to cross hybridise with other insect synapsin proteins, as a parallel synaptic marker [85].

Our results showed a distribution of honeybee neurexin I and synapsin that is broadly similar to the pattern described for *Drosophila* (Zeng et al. [65]; Figure 8). Honeybee neurexin I expression generally appears to be associated with the neuropil of the brain (Figure 8) encompassing dendrites, axons and glial processes but excluding neuronal cell bodies. The expression of bee neurexin I is thus distinct from the expression of the *neurexin I* transcript in cell bodies, as shown by the *in situ* hybridisation data, and is broadly similar to the expression pattern of other insect synaptic proteins [65], [86]. Furthermore, the expression of bee neurexin I protein in the mushroom body neuropil is consistent with the localisation of the transcript, given that the arrangement of Kenyon cell processes are known to extend into the mushroom body calyx neuropil and lobes [77], [78]. Neurexin I localisation in the optic and antennal lobe neuropils is also consistent with the transcript data, seen juxtaposed with observed mRNA expression in cell bodies associated with the optic lobes and antennal lobes (Figure 8).

Neurexin I expression was also detected in several solitary thin neural processes which showed clear and relatively strong fluorescence scattered throughout the brain (Figure 8). For example, immuno-reactive neurites between the inner surface of the calycal neuropile and the Kenyon cell somata clusters were seen in the mushroom bodies. These are typical of the extrinsic neurons of the mushroom body which project into the calyx [77]. In the optic lobes, immuno-reactive neurites were found arranged in a rather regular, radial pattern suggesting bee neurexin I expression may be found in either centripetal or centrifugal optic lobe interneurons [77].

Some differences in the localisation of honeybee neurexin I, distinct to synapsin, were also identified. Neurexin I expression was detected in irregularly arranged, intensively fluorescent puncta within the optic lobe stratum (Figure 8), possibly suggesting a special role for neurexin I in the visual system of the bee. Given synapsin is a vesicular protein found at the synapse, the more widespread expression of neurexin I suggests it is expressed beyond the synapse. This is not surprising given the broad range of neurexin I isoforms which have been detected in the bee (e.g. membrane and putatively soluble) with potentially distinct migration modes and functions.

## Discussion

The eumetazoan synapse is thought to have evolved over one billion years ago [87]. Many pre-existing proteins were recruited for their various functional properties (adhesion, catalysis) to form synaptic proteins such as neuroligins [88] and provided the molecular foundations for the evolution of the central nervous system synapse. Our study of honeybee *neurexin I* and the *neuroligins* confirms significant levels of conservation exist between insect and vertebrate synapses. An equivalent number of *neuroligins* are found in humans and honeybee, and together they form an orthologous group that diverged from an acteylcholinesterase-type molecule in a sea squirt-like ancestor (Claudianos et al. unpublished). Both vertebrate and invertebrate neuroligins are single type-1 trans-membrane proteins that typically contain a cholinesterase domain and C-terminal PDZ binding motif. Honeybee neurexin I also shares a deep-rooted common origin and structural topology with the vertebrate neurexins. The exceptional levels of intron/exon splice site conservation, giving rise to similar neurexin and neuroligin isoform patterns between vertebrate and honeybee, suggests that the function of these molecules may essentially remain the same.

We may not have recovered all of the splice variants of *neuroligin 3* that the honeybee normally produces, but the five splice variants we have recovered already represent a greater diversity of splice forms than those currently reported for the vertebrate *neuroligins*. Some of the honeybee variants are truncated forms missing whole exons which encode domains deemed important to the regulation of binding with α-neurexins [16]. Interestingly, some of the bee variants mimic alternatively spliced transcripts found in individuals affected by autism [63]. These splicing patterns raise interesting questions as to how these alternate isoforms function in the honeybee considering their vertebrate counterparts appear to have detrimental effects.

The alternative splicing of *neurexin I* in the honeybee generates even greater diversity. Ten alternatively spliced transcripts of *neurexin I* arise from twelve alternative sites. Conservation of these sites, to sites used for alternative splicing in the human neurexins [89] suggests that there is some parallel constraint in the two systems. On the other hand, novel honeybee splice variants of *neurexin I,* unreported in vertebrates, suggest the possibility of some differences in functional roles for neurexin I in the bee. The honeybee has two predominant types of isoforms arising from two alternate 3′ exons. Both types encode C-terminal regions containing a typical trans-membrane domain and PDZ binding motif. In the vertebrate lineage, α-neurexins consist of three “LNS-EGF-LNS” repeats, while β-neurexins possess a single LNS domain, the two types arising from the use of two alternate promoters [18]. In this regard we also see *neurexin I* transcripts in the bee with varying patterns of “LNS-EGF-LNS” repeats, including α-neurexin types with three classical repeats and those which encode a single LNS domain as for vertebrate β-neurexin. However, we also identified transcripts with two LNS-EGF-LNS repeats. We speculate that the presence of alternate 3′ regions maintains extensive *neurexin* splicing diversity that otherwise arises from a separate α- and β- promoter reported in vertebrates. Moreover, the complexity discovered by honeybee *neurexin I* could perhaps represent a greater evolutionary investment in a single gene among invertebrates that is offset by multiple *neurexins* in the vertebrate lineage.

Surprisingly we also identified splice variants of *neurexin I* in the bee which give rise to putative soluble isoforms. Some earlier vertebrate studies similarly support the existence of putative soluble neurexin isoforms [70], raising the possibility that there are other integral roles for these molecules which to date remain unexplored. Such molecules, including truncated forms of neurexin I and neuroligin (i.e. mimicking autism variants, missing EF metal binding motifs), could serve regulatory roles by attenuating the function of full-length isoforms or behave as soluble signalling molecules [63], [70]. These molecular observations in the honeybee arguably suggest a diverse range of neuroligin-neurexin interactions that need to be either confirmed to be similar or absent in vertebrates. Extensive life stage sampling and labour intensive cloning and sequence characterisation of RNA products remains the only effective tool. Understanding precise mechanisms of binding and oligomerisation, including what isoform combinations contribute to heterophilic and homophilic interaction between neurexin I and the neuroligns, remains a high priority.

The RNA and protein localisation data leave us with a number of questions concerning synaptic connectivity in the insect brain. Antibody staining shows neurexin I is associated with the surrounding neuropil, which largely represents dendritic arborisation including the calycal cups of the mushroom body. This neurite association overlays the expression of other synaptic proteins such as synapsin and *Drosophila* Nrx1 [65]. *In situ* RNA staining confirms *neurexin I* and *neuroligin* expression is associated with closely neighbouring cell bodies throughout the brain. We also see an intriguing differential expression pattern between *neurexin I* and *neuroligin-3* juxtaposed in subpopulations of Kenyon cells in the mushroom body. Neurexin I and the neuroligins may thus provide important functional markers for analysing neural circuitry in the bee brain. As reported by a number of studies, neuroligins and neurexins have a role in memory and learning defects that result in documented neurological disorders such as autism. Neuroligin and neurexin gene knockout studies contrast a high molecular conservation with the lack of an obvious role in early development. We therefore postulate that the neuroligin/neurexin complex is selectively constrained through evolution because it primarily participates in post-natal and/or adult sensory synaptic plasticity. The honeybee is a sophisticated behavioural model with which to study learning and memory including sensory processing [47]. We have shown these molecules have a high evolutionary conservation spanning hundreds of millions of years and thus establish an important prerequisite for using the bee as a model for vertebrate synaptic development. Dissecting the role neuroligins and neurexin I play in the bee brain is a challenge worth accepting.

## Materials and Methods

### Bioinformatics

Putative *neurexin* and *neuroligin* genes were identified in the *Apis mellifera* (honeybee) genome by homology-based searches using the BLAST tools from NCBI (http://www.ncbi.nlm.nih.gov) and Beebase (Honeybee Genome Database by Baylor College of Medicine at http://racerx00.tamu.edu/blast/blast.html). The honeybee *neuroligin* sequences were assembled using homology-based searching of the honeybee genome with the *Drosophila* (CG31146; CG13772; CG34127; CG5030) and *Anopheles gambiae* (mosquito) (COEnrl2B; C40C9.5; COEnrl1B; COEnrl8o; COEnrl16o) *neuroligin* sequences. Similarly, to assemble putative honeybee *neurexin* sequences, *Drosophila neurexin* sequences (CG7050, CG6827) were used in homology-based searches.

In conjunction with the homology-based searches, putative gene predictions were assembled together with information from predicted *in silico* sequences provided by Beebase, albeit the homology-based gene predictions produced sequences with higher sequence identity to characterised vertebrate *neuroligin* and *neurexin* genes. Sequence from the following seven Beebase (GB) gene predictions partially matched the homology-based gene predictions found from using the respective *Drosophila* (CG) and mosquito (CO) *neuroligin* sequences: (1) GB18720/CG31146/COEnrl2B; (2) GB10066/CG13772/C40 C9.5; (3) GB18290/CG34127/COEnrl1B; (4) GB18836/CG34139/COEnrl8o; (5) GB13939/COEnrl16o; (6) GB18754/CG7050; (7) GB14382/CG6827. BLAST analysis helped identify the chromosomal position and gene arrangement of the cloned honeybee transcripts. Sequence analysis tools SPIDEY [59], ExPASy [67], PROSITE [67], SMART [90], [91] and CDART [92] helped predict protein motifs of the neuroligins and neurexins (outlined in Supplementary Data). ClustalW [93] multiple protein alignments were used to construct phylogenetic trees as determined by the Neighbor Joining method with bootstrap re-sampling (detailed in the legends for Figures 1), using MEGA3.1 [94]. Congruent tree topology was observed using maximum parsimony analysis [94].

### Homology Modelling

The sequences of AmNLG1, AmNLG3 and the mouse NLG1 sequence [51], were downloaded from Swiss Prot [95] (accession number Q99K10) and were aligned using the T-COFFEE server [96]. Using these alignments, the sequences were threaded onto the templates using the program DEEP VIEW [97]. Preliminary models were then optimized using the SWISS-MODEL workspace [98], which succeeded in ligating all loops and minimizing the structure. The robustness of the structure was monitored through analysis of plots of the Anolea mean force potential [99] and GROMOS [100] empirical force field energy, as implemented in the workspace. All structures were of good quality, consistent with the quality of the alignments.

### Brain and Tissue Dissection and RNA Extraction

All bees used in these analyses were worker females. Larval samples were collected 5 days after hatching and pupae at stage P8, as per established developmental criteria in Ganeshina et al [101] (see Table 1). To collect adult bees, a single brood frame was obtained from the hive and placed in an incubator at 32°C (80% humidity). Newly emerged adult individuals were collected within 5 minutes of emerging from their cell. Bees were used immediately for dissection of fresh tissue, or caged and returned to an incubator at 32°C (80% humidity) for harvesting at days 3 and 7 post emergence. Forager bees that typically carry pollen aged between 21 to 35 days were captured near the hive entrance and similarly used for fresh tissue. Bees were cold anesthetised and brain tissue dissected. The head was separated from the body and frontal section of the head capsule removed to reveal the brain. The exposed brain was placed in diethyl pyrocarbonate (DEPC) treated water, glands removed and brain lifted from head capsule and placed in a tube on dry ice for immediate RNA extraction.

Total RNA was isolated form 10–20 frozen brains using the Trizol reagent method (Invitrogen Life Technologies). Adult brain RNA pellets were resuspended in 20–60 µl of distilled water, depending on the downstream application and desired concentration. RNA extraction from whole larvae and adult wings, legs, thoraces and abdomens was performed with the same protocol as above, with the following details; RNA from two larvae was resuspended in 100 µl of distilled water. RNA extraction from the wings, legs, thoraces and abdomens of 10 bees was pooled and resuspended in 60 µl of distilled water. Total RNA used for RT-PCR amplification was treated with DNase I (Invitrogen: #18068-015). 2–4 µl of this RNA was then used for gel electrophoresis to assess the integrity of the extraction using a 1.5% Tris-Acetate-EDTA (TAE) gel made with DEPC-treated water and run in DEPC-based TAE buffer. RNA samples used for quantitative real time PCR were then quantified by spectrophotometry using a Nanodrop (Biolab: # ND1000; V3.2 software).

### General Molecular Methods

The honeybee *neuroligin* and *neurexin I* transcripts were identified by RT-PCR amplification using the SuperScript III One-Step RT-PCR System with Platinum® *Taq* DNA Polymerase (Invitrogen: # 12574-018/026). Table S1 (Supplementary Data) outlines the primer sets (synthesised by Geneworks, Australia), cDNA samples and PCR conditions used for amplification of each gene. (*Eco*RI sites were designed in the primers). RT-PCR amplicons were visualized using low melt TAE agarose gels and desired bands extracted for 10 µl in-gel ligation reactions performed overnight at 16°C, with the cloning vector pGEM®-T Easy (Promega: # A1380). 50 µl of distilled water was then added to each reaction and competent JM109 *E.coli* cells (Promega: #L2001) were chemically transformed with 10 µl of the diluted ligation reaction as per standard methods and grown overnight at 37°C on LB (Luria broth) agar media supplemented with ampicillin using standard (X-Gal/IPTG) blue/white selection. Single *E.coli* colonies were used to inoculate 10 ml of LB with 20 µl of 50 µg/µL ampicillin, and grown overnight at 37°C shaking. The plasmids were recovered using the QIAprep Spin Miniprep Kit (Qiagen: # 27104). Diagnostic restriction digests with *Eco*RI [102] were performed to check the fidelity of ligation. Plasmid DNA was then quantified by spectrophotometry with a Nanodrop (Biolab: # ND1000; V3.2 software) and sent for sequencing with M13 universal primers by Micromon Services (http://www.micromon.monash.org/). Gene specific primers were then designed to sequence longer amplicons.

### 3′RACE

Nested 3′RACE (Rapid amplification of cDNA ends) primers used to confirm exon 28 in *A.mellifera neurexin I* (*AmNrxI-B)* are outlined in Table S1. Total RNA was isolated from the brain tissue of a 3 day old bee and treated with DNase I using the RNAqueous® -Micro Kit, following the manufacturer's instructions (Ambion). First strand reverse transcription reactions were carried out following the manufacturer's instructions using SuperScript™ III Reverse Transcriptase (Invitrogen). The 3′ RACE cDNA synthesis reaction was primed with the 3′ RACE Adapter (Table S1). Additionally, positive and negative control reactions with oligo (dT)_18_ were performed. The negative control contained no reverse transcriptase. The control reactions were performed with the (dT)_18_ primed cDNA reactions using primers specific for *Synaptotagmin* in *A.mellifera,* SYT-F and SYT-R (Table S1).

1 µL of template was used for each PCR with 1.5 U Taq polymerase (Fermentas) in 50 mM KCl, 3 mM MgCl2, 10 mM Tris HCl (pH 9), 0.08% Nonidet P40 and 0.4 mM of dNTP's in a 25 µl volume. PCR conditions used an initial denaturation (5 m at 95°C), followed by 30 or 35 cycles (30 s at 95°C, 45 s at 52°C or 55°C, 2 m at 72°C). For the first round of amplification of the 3′ end, the cDNA reaction primed with the 3′RACE Adapter (Table S1) was used as template and 3′RACE Outer Primer and AmNrx1ex28_5′_2 were the primers (Table S1). These PCR reactions were performed for 35 cycles with an annealing temperature of 52°C. For the second round of amplification of the 3′ end, primers internal to the first round PCR product were used. The first round PCR was used as template with the 3′RACE Inner Primer and AmNrx1ex28_5′_3 as the primers (Table S1). These PCR reactions were performed for 30 cycles with an annealing temperature of 55°C. PCR products appearing in the second reaction were separated by agarose electrophoresis and cloned into pGEM-T Easy (Promega) and sequence verified. The SYT positive control reaction generated the expected product of 200 bp, and the negative control yielded no product. Analysis of the sequences confirmed a 3′ untranslated region, spanning 870 bp from the (exon 28) stop codon TAG to the polyA tail.

### Quantitative Real Time PCR (qRT-PCR) Amplification

Primers used to analyse the expression of honeybee *neuroligins* and *neurexin I* were manually designed and then verified by PRIMER [103]. All primers were designed to work at similar annealing temperatures and to generate similar sized PCR amplicons. The primer sets were first checked with standard PCR amplification and gel electrophoresis (2.5% TAE, 25 bp ladder Promega: #19928601), and then used in a test qRT-PCR experiment to assess primer specificity. All primer sets were highly gene-specific and produced a single dissociation/melting (T_m_) curve. Positive control reactions used primer sequences for the housekeeping gene *Ribosomal Protein L8* (*RPL8)* that has been shown to be the best correlate with RNA concentration across varying honeybee developmental life stages and tissues [104]. Table S1 outlines the primers sets that were used, all at an annealing temperature of 55°C. Brain dissections and total RNA extraction were as outlined above. 1 µg RNA was used in a 20 µL cDNA synthesis reaction, using the IScript cDNA synthesis kit (Bio-Rad #170-8891). cDNA samples were then used at a dilution of 1∶20. The qRT-PCR reactions were set up with an automated liquid handling instrument (Beckman Coulter: Biomek® 3000) into 96 well PCR plates (Bio-Rad: #2239441), and performed in triplicate. Each reaction was 25 µLs in total volume, composed of 10 µL 1∶20 cDNA sample and 25 µL master mix (12.5 µL ITaq SYBR Green Super-mix with ROX (Bio-Rad: #170-8850), 0.5 µL 10 µm forward primer, 0.5 µL 10 µm reverse forward primer, 1.5 µL water). Each master mix was prepared immediately before the experiment and kept in darkness as much as possible. qRT-PCR amplification was performed by the ABI Prism® 7000 Sequence Detection System (Applied Biosystems, 7000 SDS Instrument), Version 1∶2∶3. The relative quantification (ddCt) assay default settings were used, with the addition of an extra 15 second annealing step at 55°C. Relative quantification and standard deviation calculations were derived by the comparative method (outlined by Applied Biosystems). Methodology for final data analysis and the presentation of results was taken from Collins et al [104], whereby expression levels were normalised by subtraction against the threshold cycle of *RPL8*.

### *In situ* Hybridisation

The protocols for *in situ* hybridisation were taken from Vidovic et al. [86]. Detailed methodology and modifications to the published protocol are provided in supplementary data (Materials and Methods S1). Six brain sections were assayed for each gene category.

### Immunohistochemistry

Adult worker bees were caught at the hive entrance, anesthetized by cooling and sacrificed by decapitation. Brains were dissected free and fixed in 4% freshly depolymerised paraformaldehyde in 0.1 M phosphate buffer (pH 7.3). After thorough rinsing in the same buffer, the samples were cryoprotected with 10% sucrose, frozen and sectioned at 5 µm. The polyclonal (rabbit) antibody against neurexin-I was kindly provided by Dr Wei Xie, Department of Genetics and Developmental Biology, Genetics Research Center, Southeast University Medical School, China. Monoclonal antibody against *Drosophila* synaptic-vesicle associated protein synapsin I (SYNORF1) was purchased from the Developmental Studies Hybridoma Bank (DSHB), USA. For immuno-staining, sections were permeablized with 0.2% Triton X100 in 0.1 M PBS, pre-incubated with 2% normal goat serum for one hour and incubated with either SynOrf-1 (dilution 1∶50) or neurexin-I antibody (dilution 1∶20) overnight at 4°C. Multiple rinsing in 0.1 MPBS was followed by incubation with secondary antibodies diluted at 1∶300 for one hour at room temperature. Alexa-546-conjugated anti-rabbit antibody (Molecular Probes, Invitrogen) was applied in the case of Neurexin-1, and Alexa-488-conjugated anti-mouse antibody (Molecular Probes, Invitrogen) was applied in the case of SynOrf-1. After thorough rinsing, sections were mounted in 50% glycerol. For neurexin-I and SynOrf-1 double labelling; sections were incubated in a mixture of primary and secondary antibodies mentioned above. In control experiments, primary antibodies were omitted. Digital images were taken in a Zeiss Axioscop fluorescent microscope using SPOT digital camera with SPOT RT software. The images were optimized by adjusting brightness/contrast levels using Adobe Illustrator software. Six brains were assayed.

## Supporting Information

Table S1 (2.26 MB DOC)

Figure S1 Honeybee Neuroligin 2 (AmNLG2) Black represents translated RT-PCR amplified sequence-approximately 65% of the full gene sequence. Grey represents putative sequence provided by GB10066. Blue represents the predicted start region derived from homology-based analysis.
(0.33 MB TIF)

Figure S2 (0.17 MB DOC)

Figure S3 (0.06 MB DOC)

Figure S4 (0.10 MB DOC)

Figure S5 (0.11 MB DOC)

Figure S6 (0.10 MB DOC)

Figure S7 (0.11 MB DOC)

Figure S8 (0.07 MB DOC)

Figure S9 (0.15 MB DOC)

Materials and Methods S1 (0.14 MB DOC)
