# Supplementary material for: Bridging the Synaptic Gap: Neuroligins and Neurexin I in Apis mellifera
Source: PLoS One. 2008 Oct 31;3(10):e3542. doi: 10.1371/journal.pone.0003542 (PMC2570956; doi:10.1371/journal.pone.0003542)
Supplement: Table S1 — (2.26 MB DOC) [file pone.0003542.s001.doc]

**Table S1: Primers**

**Table S1: (continued)**
