# Supplementary figures and images for: Bridging the Synaptic Gap: Neuroligins and Neurexin I in Apis mellifera
Source: PLoS One. 2008 Oct 31;3(10):e3542. doi: 10.1371/journal.pone.0003542 (PMC2570956; doi:10.1371/journal.pone.0003542)

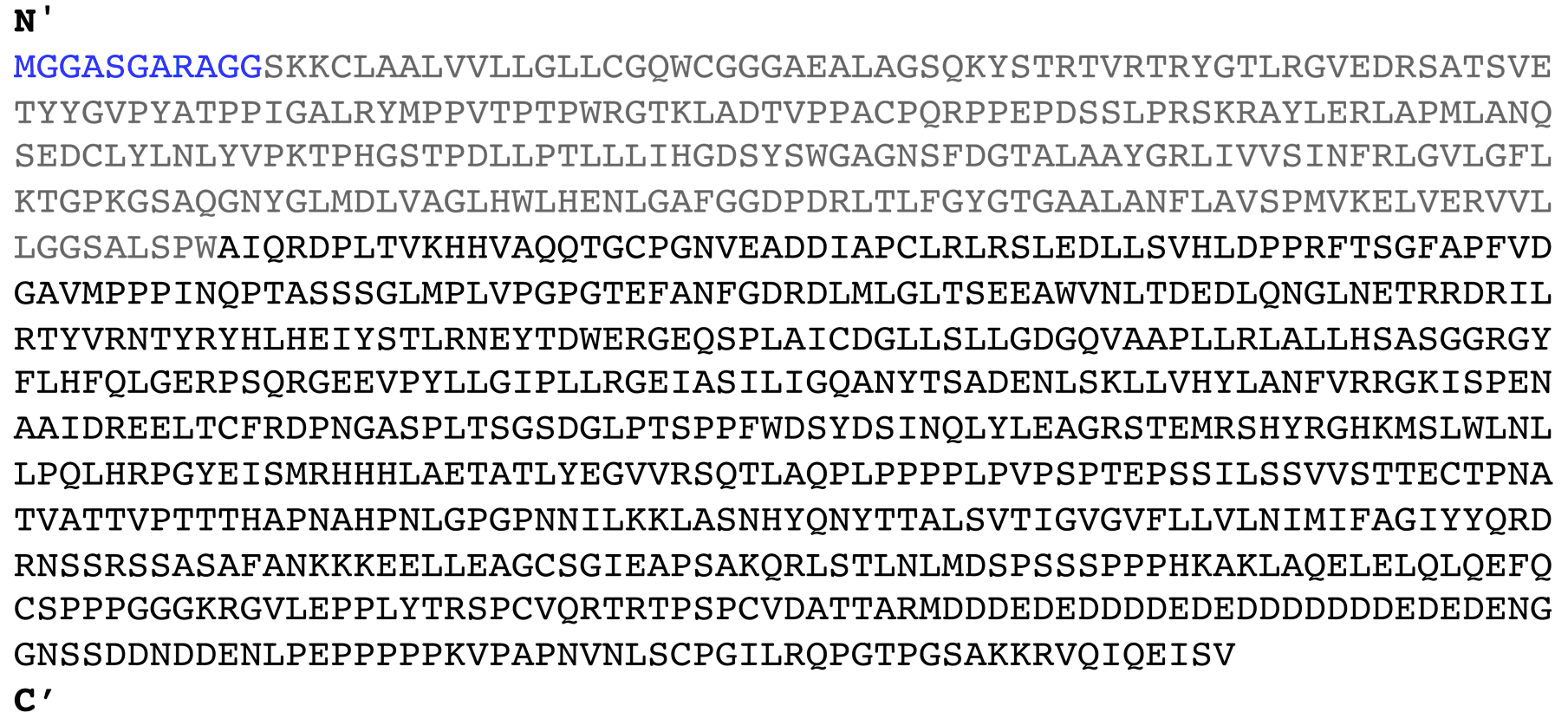

Supplement: Figure S1 — Honeybee Neuroligin 2 (AmNLG2) Black represents translated RT-PCR amplified sequence-approximately 65% of the full gene sequence. Grey represents putative sequence provided by GB10066. Blue represents the predicted start region derived from homology-based analysis. (0.33 MB TIF) [file pone.0003542.s002.tif]
