# Supplementary material for: Bridging the Synaptic Gap: Neuroligins and Neurexin I in Apis mellifera
Source: PLoS One. 2008 Oct 31;3(10):e3542. doi: 10.1371/journal.pone.0003542 (PMC2570956; doi:10.1371/journal.pone.0003542)
Supplement: Figure S2 — (0.17 MB DOC) [file pone.0003542.s003.doc]

**Figure S2: Neuroligins and Acetyl cholinesterase Multiple Alignment**

Figure S2: Alignment of the Honeybee, *Drosophila*, *C. elegans* and Human neuroligins (NLG) with Acetyl cholinesterase (*Torpedo californica*). The amino acid sequences of human, *C.elegan* and *Drosophila* neuroligins with the *Torpedo californica* acetyl cholinesterase (AChE) were taken from NCBI. The honeybee sequences were RT-PCR amplified, cloned, sequence confirmed and translated using the EXPASY tool. The cloned and sequenced-confirmed AmNLG2 sequence proceeds from the underlined sequence “CPGNVEA” to the stop codon. Sequence 5' to “CPGNVEA” was predicted based on orthology to paralogues and orthologues. All other honeybee neuroligins were cloned in full, from start codon to stop codon. The multiple alignment was performed using MEGA3.1, and exported into ClustalW. Intron/exon splice junctions are shown by red stars, and were deciphered by NCBI BLAST analysis against genomic DNA. Stop codons are depicted by asterisks. The *Drosophila* CG31146 stop codon is depicted by a carat symbol (^) highlighting a predicted stop codon. Structural motifs are depicted directly above the corresponding amino acid sequences. The signal peptide and trans-membrane domain were taken from Ichtchenko et al. [15] and Philibert et al. [111]. The EF hand metal binding motifs were taken from Tsigelny et al. [112]. The PDZ binding motif was taken from Irie et al [22]. Sequence depicted as the carboxyl/cholinesterase domain was found using the NCBI Conserved Domain Architecture Retrieval Tool. The nine conserved cysteines of the neuroligins are highlighted by grey shading [55]. Grey arrow heads highlight the last four of these nine cysteines which are not found in invertebrates but are present in the vertebrate neuroligins. Purple arrows above the amino acid sequence highlight the eight cysteines which make up four characterised disulfide bridges in human neuroligin one [10]. These arrows are numbered 1-4 to indicate which cysteines make up disulfide bridge 1 to disulfide bridge 4, respectively. The second disulfide bridge exists in sequence which undergoes alternative splicing- shown by grey lettering. The three critical and structural disulfide bridges characterised in AChE [113] are highlighted with numbered dark blue arrows from below the amino acid sequence. These arrows are numbered 1-3 to indicate which cysteines make up disulfide bridge 1 to 3, respectively. The two sites of O-linked glycosylation characterised in human neuroligin 1 (S683 and S686) are each highlighted in red and bold, and are numbered below the amino acid sequences. Alternative threonine and serine residues, which may be potential sites of O-linked glycosylation, are shaded in grey. The four sites of N-linked glycosylation in human neuroligin 1, N109, N303, N343 and N547 [10] are each highlighted in red and bold, and are numbered below the amino acid sequences. N303 occurs in sequence which undergoes alternative splicing shown in grey lettering. Oligermerisation residues characterised in human NLG1 [33] conserved in other genes shaded pink. The critical catalytic triad of AChE is highlighted by light blue shading of S/E/H and a light blue arrow head below the corresponding amino acid sequence. The ‘GESAG’ pentapeptide of the AChE active site gorge is highlighted by bold, dark blue lettering. The oxyanion hole of the AChE active site gorge is highlighted by boxed residues G/G/A. The three substrate binding sites of AChE are underlined and numbered. The 14 critical aromatic residues which line the AChE active site gorge are highlighted by yellow shading. Bold asterisks are shown below residues which have replaced by other aromatic residues in some of the neuroligins. Residues which create the salt bridges in AChE are highlighted by green shading. Numbers 1 to 4 are shown below these residues to indicate which residues make up salt bridges 1 to 4 respectively. Mutations associated to autism spectrum disorders are shaded in black and numbered below the amino acid sequences. D396 to a stop codon, and R415C were taken from Jamain et al [39]. E418D which creates a down stream codon was taken from Laumonnier et al [41]. G99S, K378R, V403M and R704C were taken from Yan et al [42]. Residues coloured by red shading highlight neuroligins where these mutations naturally occur. Abbreviations- TMD: trans-membrane domain; PDZ: PDZ (*P*ostsynaptic density 95/*D*iscs large/*Z*ona occludens 1) binding motif; Hm: human; Am: *Apis mellifera*; Dm: *Drosophila melanogaster*; CElg: **Caenorhabditis elegans;** TAChE: *Torpedo californica* acetyl cholinesterase. NB/ numbered residues refer to the gene from which the characterisation was deciphered.
