# Supplementary material for: Bridging the Synaptic Gap: Neuroligins and Neurexin I in Apis mellifera
Source: PLoS One. 2008 Oct 31;3(10):e3542. doi: 10.1371/journal.pone.0003542 (PMC2570956; doi:10.1371/journal.pone.0003542)
Supplement: Figure S3 — (0.06 MB DOC) [file pone.0003542.s004.doc]

**Figure S3: Alternatively Spliced Honeybee Neuroligins Multiple Alignment**

Figure S3: Multiple Alignment of Human Neuroligins 1, 2, 3 with Honeybee Neuroligin (NLG) Alternate Isoforms. The human neuroligin amino acid sequences were taken from NCBI, and aligned with the honeybee neuroligin 3 alternatively spliced protein sequences using the ClustalW algorithm. The honeybee sequences were RT-PCR amplified, cloned, sequence confirmed and translated using via the EXPASY tool. Intron/exon splice junctions were deciphered by NCBI BLAST analysis against genomic DNA and are indicated above the amino acid sequences; where by two numbers specify the neighbouring exons (e.g. 1/2 marks the splice junction between exon 1 and exon 2). Three sites of alternative splicing found in honeybee neuroligin 3 are indicated above the amino acid sequences by purple arrows- notably these coincide with intron/exon splice junctions. Human neuroligin alternative splice sites A and B are indicated from below the amino acid sequences by blue arrows, with the spliced insert sequences shaded in black [15]. Structural features are depicted above the amino acid sequences. Details of the signal peptide and trans-membrane domain were taken from [15]. Details of the EF-hand metal binding motifs were taken from Tsigelny et al. [112]. Details of the PDZ-binding residues were taken from Irie et al [22]. Horizontal arrows above the amino acid sequences define the carboxyl/cholinesterase domain, deciphered by the NCBI **Conserved Domain Architecture Retrieval Tool. Grey shading of HumNLG3 and HumNLG4X highlights the respective exons which are spliced in autism affected individuals [63]**. Abbreviations - EF: EF-hand metal binding motif; P: PDZ (*P*ostsynaptic density 95/*D*iscs large/*Z*ona occludens 1) binding motif; CCD: carboxyl/cholinesterase domain; a, b, c, d and e: honeybee *neuroligin 3* transcripts a, b, c, d and e respectively; Hum: human.
