# Supplementary material for: Bridging the Synaptic Gap: Neuroligins and Neurexin I in Apis mellifera
Source: PLoS One. 2008 Oct 31;3(10):e3542. doi: 10.1371/journal.pone.0003542 (PMC2570956; doi:10.1371/journal.pone.0003542)
Supplement: Figure S4 — (0.10 MB DOC) [file pone.0003542.s005.doc]

**Figure S4: Neurexin Multiple Alignment**

Figure S4: Multiple Alignment of Honeybee Neurexin I Proteins with the Human α-neurexins, *Drosophila* and *C.elegan* Neurexin I Proteins. The amino acid sequences of the human, *C.elegan* and *Drosophila* neurexins were taken from NCBI, and aligned with honeybee neurexin I_A and neurexin I_B sequences using the ClustalW algorithm. The honeybee sequences were RT-PCR amplified, cloned, sequence-confirmed and translated using via the EXPASY tool. The twelve sites of alternative splicing found throughout the honeybee neurexin I gene are numbered and indicated below the amino acid sequences with purple pointers –some of these coincide with intron/exon splice junctions whilst others exist within exons. The five characterised sites of alternative splicing in the human neurexins are numbered and indicated from above the amino acid sequences with blue pointers [89]. The third site of alternative splicing in the honeybee coincides with the first site of alternative splicing in humans. Intron/exon splice junctions are shown by red stars, and were deciphered by NCBI BLAST analysis against genomic DNA. Structural features are depicted above the amino acid sequences. The Laminin G-like (or LNS) and EGF domains were obtained using PROSITE. PDZ domain information taken from Rissone et al. [109] for AmNrxI_A, and deciphered from Jeleń et al. [71] for AmNrxI_B. The signal peptide and trans-membrane domain were taken from Missler et al. [89]. The putative PDZ domain of AmNrxI_B, and associated residues, shown above the sequence and shaded in grey respectively. Characterised calcium binding sites shaded in blue, namely D137, V154, I236 and N238. Corresponding calcium binding sites, confirmed through homology modelling also, shaded in green, namely M145, V236, D137 and N238. Abbreviations -EGF: *E*pidermal *G*rowth *F*actor motif; PDZ: PDZ (*P*ostsynaptic density 95/*D*iscs large/*Z*ona occludens 1) binding motif; Hm: human; Am: *Apis mellifera*; Dm: *Drosophila melanogaster*; CElg: **Caenorhabditis elegans.**
