# Supplementary material for: Bridging the Synaptic Gap: Neuroligins and Neurexin I in Apis mellifera
Source: PLoS One. 2008 Oct 31;3(10):e3542. doi: 10.1371/journal.pone.0003542 (PMC2570956; doi:10.1371/journal.pone.0003542)
Supplement: Figure S5 — (0.11 MB DOC) [file pone.0003542.s006.doc]

**Figure S5: Alternatively Spliced Honeybee Neurexin I Multiple Alignment**

Figure S5: Multiple Alignment of honeybee alternate neurexin I isoforms. The honeybee alternatively spliced neurexin I amino acid sequences were aligned using the ClustalW algorithm. The sequences were RT-PCR amplified, cloned, sequence-confirmed and translated using via the EXPASY tool. Intron/exon splice sites were deciphered by Beebase BLAST analysis against genomic DNA. The intron/exon splice junctions are highlighted with red stars and labelled with two numbers above the amino acid sequence, specifying the neighbouring exons (e.g. 1/2 marks the splice junction between exon 1 and exon 2). The twelve sites of alternative splicing found throughout the honeybee neurexin I are numbered and indicated above the amino acid sequences with purple pointers –some of these coincide with intron/exon splice junctions whilst others exist within exons. The five characterised sites of alternative splicing in the human neurexins, are numbered and indicated from below the amino acid sequences with blue pointers [89]. The third site of alternative splicing in the honeybee coincides with the first site of alternative splicing in humans. The two alternate bee neurexin I stop codons are highlighted by a circled 1 and 2 above asterisks in the amino acid sequences. Structural features are depicted above the amino acid sequences, except the trans-membrane domain and the potential PDZ domain of AmNrxI_B9 -which are shown below. The Laminin G-like (or LNS) and EGF domains were obtained using PROSITE. AmNrxI_A and AmNrxI_A2 PDZ domain derived from Rissone et al. [109]. PDZ domain of AmNrxI_B variants deciphered from Jeleń et al. [71] for AmNrxI_B variants. Details about the signal peptide and of the trans-membrane domain of AmNrxI_A and AmNrxI_A2 were taken from Missler et al. [89]. The trans-membrane domain of AmNrxI_B9 was obtained using SMART analysis. Potential sites of O-glycosylation highlighted with grey shading. Calcium binding sites, confirmed through homology modelling also, shaded in green, namely M145, V236, D137 and N238. Abbreviations- LamG: Laminin G-like Domain; EGF: *E*pidermal *G*rowth *F*actor motif; PDZ: PDZ (*P*ostsynaptic density 95/*D*iscs large/*Z*ona occludens 1) binding motif; Am: *Apis mellifera*
