# Supplementary material for: Bridging the Synaptic Gap: Neuroligins and Neurexin I in Apis mellifera
Source: PLoS One. 2008 Oct 31;3(10):e3542. doi: 10.1371/journal.pone.0003542 (PMC2570956; doi:10.1371/journal.pone.0003542)
Supplement: Figure S6 — (0.10 MB DOC) [file pone.0003542.s007.doc]

**Figure S6: Honeybee NLG1 and Mouse NLG1 Alignment**

AmNLG1 TREVRVKQGRLRGIVVQPRTNHDLQPVDVFLGVPYAEPPVNFLRFSPPRSPEPWRGTRES

MmNLG1 52 DPLVTTNFGKIRGIK-KELNNEILGPVIQFLGVPYAAPPTGEHRFQPPEPPSPWSDIRNA 110

AmNLG1 QEFAPVCPQVV--PKLQDEMKPVRY-EYLERLLPYLKNQSEDCLYLNIYTPHQPEGQKTL

MmNLG1 TQFAPVCPQNIIDGRLPEVMLPVWFTNNLDVVSSYVQDQSEDCLYLNIYVPTEDGGPK-- 193

AmNLG1 RKYPVMVFIHGESFEWNSGNPYDGTILAAYGNVVFVTISFRLGILGFLRPGIRDDTASNF

MmNLG1 ---PVMVYIHGGSYMEGTGNLYDGSVLASYGNVIVITVNYRLGVLGFLSTG-DQAAKGNY 249

AmNLG1 GLLDQIAALLWLRENIAEFGGDPNSITLVGHGTGAIFANLLLISPVANKKGLFRRAILMS

MmNLG1 GLLDLIQALRWTSENIGFFGGDPLRITVFGSGAGGSCVNLLTLSHYS--EGLFQRAIAQS 316

AmNLG1 GSALSADAIGKAPLQITKQVAHALHCPTTTDSDLAICLRGQDVDTLLNVKIHKPSYVPAF

MmNLG1 GTALSSWAVSFQPAKYARILATKVGCNVSDTVELVECLQKKPYKELVDQDVQPARYHIAF 376

AmNLG1 APLIDNAVIPDKPYNLMKNPQMFDRFDQMYGVTESEKYHLL-SPVDLMHGMSEGQRDAVL

MmNLG1 GPVIDGDVIPDDPQILMEQGE-FLNYDIMLGVNQGEGLKFVENIVDSDDGVSASDFDFAV 435

AmNLG1 KEHAKATHELEAELILSKILEQYGDFSPGFQGEYMLKNRDLVLEALSDSGTAAPLIMAAN

MmNLG1 SNFVDNLYGYPEGVLRETIKFMYTDWADRHNPE---TRRKTLLALFTDHQWVAPAVATAD 494

AmNLG1 LHSRANPNSYMYVFAHPKATQEYSGQQRKYTVHSEELPYLLGAPLDG----LRGRYDIGG

MmNLG1 LHSNFGSPTYFYAFYHHCQTDQVPAWAD--AAHGDEVPYVLGIPMIGPTELFPCNFSKND 552

AmNLG1 TLFSEAIMNWWCSFAYIGNPNVKAISKRYPYMRNGLKELSQYDIDWPEYDPQNQRYLNLT

MmNLG1 VMLSAVVMTYWTNFAKTGDPNQ-PVPQDFE------------EVAWTRYSQKDQLYLHIG 609

AmNLG1 IPPSTGMQYRLAEMQFWSEHLPKMLL

MmNLG1 LKPRVKEHYRANKVNLWLELVPHLHN 635

Figure S6: Honeybee neuroligin 1 (AmNLG1) and mouse neuroligin 1 (MmNLG1) alignment. The mouse neuroligin sequence (accession number Q99K10) was taken from Swiss-Prot and aligned with honeybee neuroligin 1 using the ClustalW algorithm for T-COFFEE, version 5.53. The cysteine residues involved in disulfide bridges (mouse numbering: C117-C153, C342-C353 and C512-C546) are highlighted in blue. The two helices (450-460 and 620-635) that constitute the neuroligin dimerisation domain are shown in red font. The neuroligin dimerisation residues characterised in mouse are highlighted with grey shading (W626, L625, L629 W463, F458 and M459), as are conserved hydrophobic residues in AmNLG1. The residues at each end of the neurexin interface domain are shaded in red (D387 and D402). Central residues of neurexin interface domain shaded in black (Q395N, G396P, E397Q, F398F, N400R, F499A).
