# Supplementary material for: Bridging the Synaptic Gap: Neuroligins and Neurexin I in Apis mellifera
Source: PLoS One. 2008 Oct 31;3(10):e3542. doi: 10.1371/journal.pone.0003542 (PMC2570956; doi:10.1371/journal.pone.0003542)
Supplement: Figure S7 — (0.11 MB DOC) [file pone.0003542.s008.doc]

Figure S7: Honeybee NLG3 and Mouse NLG1 Alignment

MmNLG1 52 DPLVTTNFGKIRGIKKELNNEILGPVIQFLGVPYAAPPTGEHRFQPPEPPSPWSDIRNAT 111

AmNLG3 -RIVRTKYGDLSGVIVTLDRHLEG-VEVFRGVPYASPPIGSLRFMPPVSSALWHGVKVAD

MmNLG1 112 QFAPVCPQNIIDGRLPEVMLPVWFTNNLDVVSSYVQDQSEDCLYLNIYVPTGP-------

AmNLG3 KFGPVCPQ--RLPELSDKMP-KGRVEYLRRLLPYLRNQSEDCLYLNVYAPVQAGARDGGG

MmNLG1 193 --KPVMVYIHGGSYMEGTGNLYDGSVLASYGNVIVITVNYRLGVLGFLSTG---DQAAKG

AmNLG3 RRYPVIVFVHGESYEWSSGNPYDGSVLASYGGVVVVTINYRLGILGFLNANTDSHLRSPA

MmNLG1 229 NYGLLDLIQALRWTSENIGFFGGDPLRITVFGSGAGGSCVNLLTLSHYSEKG-LFQRAIA

AmNLG3 NYGLMDQIAALHWVQENIGNFGGDPRNVTLIGHGTGAACVNFLMTSHAVPDGLLFHRSVL

MmNLG1 315 QSGTALSSWAVSFQPAKYARILATKVGCN--VSDTVELVECLQKKPYKELVDQDVQPARY

AmNLG3 MSGSALSPWALVRGAANYALQVAKHLNCSWAASDSQALLRCLREVPLNALVSVPVKGLEF

MmNLG1 373 HIAFGPVIDGDVI-PDDPQ----------------ILMEQ---GEFLNYDIMLGVNQGEG

AmNLG3 APAFGPSVDGVVIDPGDPEDQDFTLQVDTINTLNNILLRKDVVAKLSRYDLMIGVVRSEA

MmNLG1 413 LKFVENIVDSDDGVSASDFDFAVSNFVDNLYGYPEGKDVLRETIKFMYTDWAD-RHNPET

AmNLG3 Y-FALTADDAQYGIEADRRTKILREFVRNTYTYHQAE--ILATIINEYTDWERPVQHPVN

MmNLG1 472 RRKTLLALFTDHQWVAPAVATADLHSNFGSPTYFYAFYHHCQTDQVPAWADAAHGDEVPY

AmNLG3 IKDETLEALGDANTVAPATRTADLHSQSRRNSYLYVFDYQSKFGDYPQKPGCIHGEDLPY

MmNLG1 532 VLGIPMIGPTELFPCNFSKNDVMLSAVVMTYWTNFAKTGDPNQPVPQDTKFIHTKPNRFE

AmNLG3 FFGAPLVGGLSHWPKNYTRAEMALSESVILYLTNFARTGNPNEGTP-DVGPMRPERTKLK

MmNLG1 592 EVAWTRYSQKDQLYLHIGLKPRVKEHYRANKVNLWLELVPHLHN

AmNLG3 NIDWIAYETVHKKYLSIELKSKLKNHYRAHRLSFWLNLVPDLH-

Figure S7: Honeybee neuroligin 3 (AmNLG3) and Mouse neuroligin 1 (MmNLG1) Alignment. The mouse neuroligin sequence (accession number Q99K10) was taken from Swiss-Prot and aligned with honeybee neuroligin 3 using the ClustalW algorithm for T-COFFEE, version 5.53. The sixteen, nine and three amino acid insertions of AmNLG3 are underlined. The 3 amino acid insertion is in a loop that, in MmNLG1, interacts with β-neurexin 1. The cysteine residues involved in disulfide bridges (mouse numbering: C117-C153, C342-C353 and C512-C546) are highlighted in blue. The two helices (450-460 and 620-635) that constitute the neuroligin dimerisation domain are shown in red font. The neuroligin dimerisation residues characterised in mouse are highlighted with grey shading (W626, L625, L629 W463, F458 and M459), as are conserved hydrophobic residues in AmNLG3. The residues at each of the neurexin interface domain are shaded in red (D387 and D402). Central residues of neurexin interface domain shaded in black (Q395, G396, E397, F398, N400, F499).
