# Supplementary material for: Bridging the Synaptic Gap: Neuroligins and Neurexin I in Apis mellifera
Source: PLoS One. 2008 Oct 31;3(10):e3542. doi: 10.1371/journal.pone.0003542 (PMC2570956; doi:10.1371/journal.pone.0003542)
Supplement: Figure S8 — (0.07 MB DOC) [file pone.0003542.s009.doc]

**Figure S8: Honeybee NrxI_A and Mouse β-Nrx 1 Alignment**

MnNrx1β 73 HSAFAADPGHTTYIFSKGGGQITYKWPPNDRPST**R**ADRLAIGFSTVQKEAVLVRVDSSSG 134

AmNrxI_A FTGPTCNEEAAAYEFGPGKGIITYTFPPNQRPEMKKDTVALGFVTSVNDAVLVRIESAS- 59

MmNrx1β LG**D**YLELHIHQGKIGVKFN**V**GTDDIAIEESNAIINDGKYHVVRFTRSGGNATLQVDSWPV 194

AmNrxI_A SD**D**YLEIEILEGNVFAFYNMGTNDHPIGEVGVKVNDNQYHVVRFTRTGPNSTLQVDDYNL 119

MmNrx1β IERYPAG**R**QLT**I**F**N**SQATIIIGG---KEQG---QPFQGQLSGLYYNGLKVLNMAAENDAN 278

AmNrxI_A QSNHPSG**R**QLT**V**F**N**SQSTIQIGGRWNRNKGRVERPFLVLIAGLVVNGARILELAVSKDGR 179

MmNrx1Iβ AIVGNVRLVGEV- 291

AmNrxI_A VVTRGDVQLLPPG 192

Figure S8: Honeybee neurexin I A (AmNrxI_A) and Mouse β neurexin 1 (MmNLG1) Alignment. The sequence of the ligand binding domain of mouse β neurexin 1 [114]; accession number Q99K10), was taken from Swiss-Prot and aligned with honeybee neurexin I A using the ClustalW algorithm for T-COFFEE, version 5.53. The residues buried upon neuroligin binding are shaded in grey (identical with AmNrxI_A N103, P106, R232, I243, N238 and S239; highly conserved with AmNrxI_A R109K, S132A, V154M and I236V; and semi-conservative differences with AmNrxI_A (S107E, T108M, and L135S). Residues that form salt bridges are shown with red font (R109K, R232). Calcium binding residues shown in blue font (D137, V154, I234 and N236).
