# Supplementary material for: Bridging the Synaptic Gap: Neuroligins and Neurexin I in Apis mellifera
Source: PLoS One. 2008 Oct 31;3(10):e3542. doi: 10.1371/journal.pone.0003542 (PMC2570956; doi:10.1371/journal.pone.0003542)
Supplement: Figure S9 — (0.15 MB DOC) [file pone.0003542.s010.doc]

**Figure S9: *AmNrxI_B* Full Gene Annotation with 3'UTR from 3'RACE Confirmation**

atgcatccctctgtcggaaacgtgggcacgttgctgccgtcgctggtcgtgctgttgagc

M H P S V G N V G T L L P S L V V L L S

caggtcgccatgtcgttcgtgctcgagggttcggccacgagttacgcccagttcaggaaa

Q V A M S F V L E G S A T S Y A Q F R K

tggaacgcggggttgaacggcacgctggagttcgagttcaagaccgagcaggggaacggc

W N A G L N G T L E F E F K T E Q G N G

cttcttctgtacacggacgacgggggcacgtacgacttcttcgaggcgaagctcgtcgag

L L L Y T D D G G T Y D F F E A K L V E

agcgcgctcaggttgaggtacaacctgggcggcggtgcccagatcgtcacggtcggccac

S A L R L R Y N L G G G A Q I V T V G H

gatctcggggacggccactggcacaaggtgcagatcaccagatgcgcggagaacaccacg

D L G D G H W H K V Q I T R C A E N T T

ttgaccgtggacggtgtgagcgcggtctccacgtcccgtggaaaggagtttgagttcggc

L T V D G V S A V S T S R G K E F E F G

aagctggccggcaattccgacgtctacgtgggcggcatgcccagctggtacaacagcaag

K L A G N S D V Y V G G M P S W Y N S K

ctcacgttgctcgcgttgccgagcgtcatcttcgagccgaggttcaacgggttgatcagg

L T L L A L P S V I F E P R F N G L I R

aatctggtgtacgccgatggcgagaacacggtgccgaggaggcaggagatgaagagccgg

N L V Y A D G E N T V P R R Q E M K S R

gacgccaagtgcggtggatttccatgcgtggagaacgtgaagcgcaaatcggcgaggagt

D A K C G G F P C V E N V K R K S A R S

ttacgcaacatgatgacagccaatacgacggacgcctgtgaaacgcgtgatccctgtcaa

L R N M M T A N T T D A C E T R D P C Q

cacggaggaatctgcatatccaccgacagtggacccatctgcgagtgtcggtcaggcgat

H G G I C I S T D S G P I C E C R S G D

tatgagggtgcctattgtgagaaagaagcatggagcacggtgctgcctacggcgggtgct

Y E G A Y C E K E A W S T V L P T A G A

ggtgattactgggcgtgggattacaataaagcgccttccgaagcttctttcaaaggaacg

G D Y W A W D Y N K A P S E A S F K G T

gagtatctcacgatagatttgagcaaaggagatccaattttgagcacacaggaaaccgtt

E Y L T I D L S K G D P I L S T Q E T V

aatttgcaatttaaaacaaaacaaccgaatggccttttattttattccggggaaggagat

N L Q F K T K Q P N G L L F Y S G E G D

gattatctgaccatatctctaagagatggaggggtggctgtaggcatgactttggccaaa

D Y L T I S L R D G G V A V G M T L A K

gggagattagatctgcacataaagcctgttcgggtccggttcgatgataatcaatggcac

G R L D L H I K P V R V R F D D N Q W H

aggatcatcgttcacaggaaagttcaagagatatcctccatcaccagcttttgcaggctg

R I I V H R K V Q E I S S I T S F C R L

tccgccatcgtcgatgggatttacgcggaacacggccacactgcgggctcgttcacacat

S A I V D G I Y A E H G H T A G S F T H

ttggcgagcgatcgactgctggtgggcggaggcgccgacgcgagatccctccagggcgcc

L A S D R L L V G G G A D A R S L Q G A

aaggggatcaacaacttcaacggatgcctgaggaaggtcgaattcgtcgcggagggggtg

K G I N N F N G C L R K V E F V A E G V

aaaatggagttgatcgaggcggccaggagtggagccgccggtgctgccgcctggggcaaa

K M E L I E A A R S G A A G A A A W G K

atggatttccactgtcgggagccgagatcctcggacccaatcaccttcaccacgagggac

M D F H C R E P R S S D P I T F T T R D

tctcatctcgtgctgccaccttggagggcggccaaatccggtagtatatccttcaaaatc

S H L V L P P W R A A K S G S I S F K I

cgtaccaacgaacctaacggattgatcatgtacagtcgtagcggagctcatacgagcaag

R T N E P N G L I M Y S R S G A H T S K

atctgttcgcattcgaaatctttggaggtcattatatctacatatcgatctcggaagcgg

I C S H S K S L E V I I S T Y R S R K R

acctgtaaagtaaagtcatcgaagcaacgaatcgacaatggaatctggcacgatgtggca

T C K V K S S K Q R I D N G I W H D V A

cttcgtcgtgttgaacgagacggacgtgtcaccgtagacgattcaatagttgaatttcgt

L R R V E R D G R V T V D D S I V E F R

acaccaggtgattcgacgcaactagatctagatggattactgtatatcggtggtgtgggt

T P G D S T Q L D L D G L L Y I G G V G

gctccatttgcaccccttacagttcctccggtactgtggacaggtgccttgagacaaggt

A P F A P L T V P P V L W T G A L R Q G

tacgttggttgcataagggatcttgtcataaacgggcaacaaatcgatatcgctggatat

Y V G C I R D L V I N G Q Q I D I A G Y

gctcaacaacaggactctggtgctgtcaaaccagcttgtcattttcaacaatcccactgt

A Q Q Q D S G A V K P A C H F Q Q S H C

ccctcacaaccctgcatgcacggtagccactgtattgagggatggaacagatttcactgt

P S Q P C M H G S H C I E G W N R F H C

gactgcactggaacacagtacactggtcctacttgtgggaaagatgcatcaacattgcat

D C T G T Q Y T G P T C G K D A S T L H

ctgaatggaacacagcaaatgacagcattgatgccagaggattcgaaaacgcagactgaa

L N G T Q Q M T A L M P E D S K T Q T E

gaaatagtagttcgtttcaaaactacgaggccacgtggtcttcttttagccacgagttta

E I V V R F K T T R P R G L L L A T S L

gaaaacagttcggatcgattgcaactctacctcgaagagggcaaagcgaagatgctgatc

E N S S D R L Q L Y L E E G K A K M L I

catatcggggacaaagaaaagacattagttgctggacaaggtttaaatgacgacatgtgg

H I G D K E K T L V A G Q G L N D D M W

cacacactgaggttctccagaagagcaagctctttgaaattccaaatcgatgacgaagct

H T L R F S R R A S S L K F Q I D D E A

gctgtacgggccgaaacgcagcttggcaaacagagcattttggaatttcggacgctccac

A V R A E T Q L G K Q S I L E F R T L H

gtcggtggctacttgcacgcaggggaggaaatcccgcatttcgtcggccagctccagcaa

V G G Y L H A G E E I P H F V G Q L Q Q

atctggttcaacggttacccgtacctggaaatagcgcgtagctccggaaatcatcaggca

I W F N G Y P Y L E I A R S S G N H Q A

tctcatcaaggtgtaacgccgattattagagtcacagggaaattcgggaagcggaaccat

S H Q G V T P I I R V T G K F G K R N H

ccagtgcatcatcctgtgactttcacgtcaaagcacacattcgtcggtttacctgtgttg

P V H H P V T F T S K H T F V G L P V L

aaagcgtacgtggaaactaacatctatttccagttcaaaacacgcgaggcaaatggttta

K A Y V E T N I Y F Q F K T R E A N G L

attctgtacaacgccggtcgtgaacgtgattccatcgcggtcgagttagtaaacgggcat

I L Y N A G R E R D S I A V E L V N G H

attcactatgtgttcgacctcggtgatggcccggtcaggatcagagacacttcgagatcc

I H Y V F D L G D G P V R I R D T S R S

aagctgaatgatggaaagtggcacgcggtcagcatcggaaggccagctccaaaaagacat

K L N D G K W H A V S I G R P A P K R H

actctcgcagttgacgaccacgtcaccgctgtgaatagccagggtagcacgagatctgac

T L A V D D H V T A V N S Q G S T R S D

ctggatgaattttgttttatcggtggtgtggagaaatcacagtatggtcagctaccgaaa

L D E F C F I G G V E K S Q Y G Q L P K

caaatactcagcaaacatggtttcgagggatgtctcgcatcgttggatcttagtggcgag

Q I L S K H G F E G C L A S L D L S G E

agtaccaatctgatttccgacgcggtagtccctagctctttagtagaatccggttgcgat

S T N L I S D A V V P S S L V E S G C D

atgtacgccaatctgcatcctggcaaaaagtgtacacacgatgtatgctcgaatcatggc

M Y A N L H P G K K C T H D V C S N H G

acgtgcgtccaacaatggaacagttatacatgcgattgtgacatgaccagcttcacaggg

T C V Q Q W N S Y T C D C D M T S F T G

ccgacgtgtaacgaagaagcggcggcttatgaattcggaccaggaaaaggaatcattacg

P T C N E E A A A Y E F G P G K G I I T

tacacctttccgccgaatcagagaccggaaatgaagaaagacactgtggctttgggcttt

Y T F P P N Q R P E M K K D T V A L G F

gtaacaagcgtaaacgatgctgtacttgtcagaatagaatctgcttcgagcgacgactat

V T S V N D A V L V R I E S A S S D D Y

cttgagattgaaattttggagggaaatgtgttcgccttttacaatatgggcacaaacgat

L E I E I L E G N V F A F Y N M G T N D

catccaattggggttaagactggtgtttgc**tag**ttaaatttgcaagttaagattagaaat

H P I G V K T G V C - L N L Q V K I R N

ataaatatagatacttttattatggataaataaatttgaaacgtgtaaatagatatctaa

I N I D T F I M D K - I - N V - I D I -

atttttgatgttttaacacatattatcttaaagatataatatttcatagttatttaattt

I F D V L T H I I L K I - Y F I V I - F

ttaaatgaattaataacaccagtctagattgcaaattattcagttgatttaaaaattgac

L N E L I T P V - I A N Y S V D L K I D

gtaaaaatcttcgcgtaaaaattatatatttattaagtccttgttaatatataacttaaa

V K I F A - K L Y I Y - V L V N I - L K

cagattgctaaaatttttgatataattcgattaatcttgttattgtctattaaattattc

Q I A K I F D I I R L I L L L S I K L F

caaattatttcaattcgtcaagaatttgtcgtttctctaaaaaaattattatctagaaaa

Q I I S I R Q E F V V S L K K L L S R K

cgcaaaaacgcataaaattggtcgattttgcaaaaaaaaaaaaagcaataatgattgtca

R K N A - N W S I L Q K K K K Q - - L S

ttgattttcaaactgttgacgcgcacttctaaatcgatcaattttgttgtcgaaaagacg

L I F K L L T R T S K S I N F V V E K T

gtgtgatatacactcgcggatgaacgaaagggagagagagaatgtgaaaaagtaatgttt

V - Y T L A D E R K G E R E C E K V M F

aggtacggtaataaaaggaatctccttaatccttgactaaaaacgagatgcaaaacgtcc

R Y G N K R N L L N P - L K T R C K T S

gattgttgaatggtttataaggtataactttgtaaactgctcactacgagctaataactc

D C - M V Y K V - L C K L L T T S - - L

gccattttttctaaaaacgacgtaccttgaacatcatgaaatcctaaagtccgataaaat

A I F S K N D V P - T S - N P K V R - N

aaagtaaaaataaaagatgaaaattaaaaaaaaaaaaaaaaatatcgaaagaataaaatg

K V K I K D E N - K K K K K Y R K N K M

aaggagaagaaaaatgtaaaatttctcgtattcttcgttgtctattcttttatgattttt

K E K K N V K F L V F F V V Y S F M I F

aaaaaaaaaaaacctatagtgagtcgtattaattctgtgctcgc

Figure S9: *AmNrxI_B* Full Gene Annotation with 3'UTR from 3'RACE Confirmation. Gene sequence of longest B variant (with exon 28) of *neurexin I*, *AmNrxI_B,* is shown (5') from start codon ATG to (3') TAG stop codon (red). Protein translation given below, derived by EXPASY. Exon 28 shaded in grey. (NB/ this is the longer version of exon 28 found in alternatively spliced variant *AmNrxI_B9*, refer to Figure S5). The 3' untranslated region (UTR) is shown in grey lettering. 3'RACE Adapter primer sequence (see Table S1) shown in blue lettering. Polyadenylation signal (PAS) underlined.
