# Supplementary material for: Bridging the Synaptic Gap: Neuroligins and Neurexin I in Apis mellifera
Source: PLoS One. 2008 Oct 31;3(10):e3542. doi: 10.1371/journal.pone.0003542 (PMC2570956; doi:10.1371/journal.pone.0003542)
Supplement: Materials and Methods S1 — (0.14 MB DOC) [file pone.0003542.s011.doc]

**Supplementary Materials and Methods**

**S1: *In situ* hybridisation**

All equipment and materials used for *in situ* work were either cleaned with alcohol and RNase Zap (Ambion: #9780–9784), baked overnight at 180°C or autoclaved.

Digoxigenin (DIG)-labelled riboprobes were synthesised using a DIG RNA Labeling Kit (SP6/T7) (Roche: # 11 175 025 910). The probe templates were linearised by restriction enzymes that create 5’ overhangs, as outlined by the manufacturer’s protocol. For synthesis of *AmNLG3* probes, the full length *AmNLG3*_pGEMT clone was used as a template. Digestion of *AmNLG3*_pGEMT with *Bam*HI followed by transcription with T7 polymerase gave rise to a 1424bp sense DIG-labelled probe (negative control). Digestion of *AmNLG3*_pGEMT with *Nar*I followed by transcription with SP6 polymerase gave rise to a 1495bp anti-sense DIG-labelled probe. For synthesis of *AmNrxI* probes, a pGEMT construct encoding the first seven exons of *AmNrxI* (common to all splice variants) was used as a template. Amplification of this *AmNrxI* fragment was achieved with the full length *AmNrxI*_pGEMT construct as a template, and primers *AmNrxI* Forward: 5’ gaattcATGCATCCCTCTGTCGGAAACGTG 3’ and *AmNrxI*_Exon7 Reverse 5’ GCCTACAGCCACCCCTCCATCTCTTA 3’. This fragment was then cloned into pGEMT to create the recombinant plasmid *AmNrxI*_Exon7_pGEMT. Digestion of *AmNrxI*_Exon7_pGEMT with *Hind*III followed by transcription with T7 polymerase gave rise to a 936bp sense DIG-labelled probe (negative control). Digestion of *AmNrxI*_Exon7_pGEMT with *Xho*I followed by transcription with SP6 polymerase gave rise to a 980bp anti-sense DIG-labelled probe. *DNase* I treatment and precipitation by 4M DEPC-based LiCl, were performed on all probes. To assess the efficiency of probe labelling and observe residual undigested template, gel electrophoresis of the probes was performed with a 1kb marker (Invitrogen: #15615-016) and 10kb RNA Marker (Sigma: #R7020), with DEPC-treated buffer and 1.25% TAE gel. Probes were quantified by spectrophotometry with a Nanodrop (Biolab: # ND1000; V3.2 software).

Whole pupa heads of stage P8 were removed and placed in fixative (4% paraformaldehyde in DEPC treated phosphate buffer saline (PBS)) at room temperature for 4hrs. Adult honeybees were cold anaesthetised (placed at -20°C for no longer three minutes) before their heads were removed and placed in fixative. The heads remained at 4^o^C whilst each brain was individually dissected from the head capsule. Each head was embedded in wax to allow for delicate and thorough removal of the brain, to maintain whole brain morphology. Dissections were performed in PBS pH7.4 under a microscope with tweezers. Each brain was immediately place in fresh fixative at room temperature for two hours. Pupa and adult tissue was then transferred to 18% sucrose in PBS for cryoprotection, overnight at 4^o^C. The tissue samples were mounted in vinyl specimen moulds **(Tissue-Tek® Cryomold® Bioposy: #4656), and** embedded in a freezing/mounting medium (Tissue-Tek® O.C.T. Compound, ProSciTech: #IA018) on dry ice and isopentane. Tissue blocks were stored at -80^o^C and warmed to -20^o^C before sectioning.

Tissue sections were cut by cryosectioning at 20μM thickness and mounted on silane-coated slides (Labscientific: # 7801). The slides were dried overnight at 40^o^C and stored at -80^o^C. Hybridization slides were subsequently warmed to room temperature and dried at 40^o^C for 2 hours and then washed in separate dishes at room temperature in the following reagents: 5 minutes in 200ml PBS, 20 minutes in 200ml 4% paraformaldehyde, 10 minutes in 0.1% DEPC in PBS and 5 minutes in fresh PBS. The sections were then incubated for 10 minutes in 5µg/ml Proteinase K (Sigma: #P6556) in PBS, at 37^o^C in a water bath. After a rinsing for 5 minutes in 0.1% DEPC in PBS at room temperature, in a separate dish, the sections were then dehydrated with 150mls of increasing concentrations of ethanol (50%, 70%, 90% and 100%) for three minutes each. Finally, sections were wrapped in foil and dried in a fume hood for 45 minutes.

Adjacent sections from the same brain were hybridised with the sense probe to check probe specificity. Anti-sense and sense hybridisation was performed in separate boxes lined with 10ml soaked paper towel (4ml formamide, 10ml 20 x SSC, 6ml DEPC-treated water). A hydrophobic barrier was drawn around the tissue sections with a liquid blocker Super Pap Pen (Daido Sangyo). The probes were diluted to 1.0 µg/ml in pre-hybridisation buffer described in [102]. The probe mix was heated for 30 seconds at 95^o^C, and briefly vortexed and spun down. 150µl of probe mix was added per section and placed on a hot plate at 95^o^C for 4 minutes. Hybridisation was carried out at 55^o^C overnight. Sense and anti-sense experiments were washed in separate dishes. They were rinsed twice in PBS, for 5 minutes at room temperature. They were digested with 10µg/ml RNaseA (Qiagen: # 19101) in PBS for 30 minutes at 37^o^C and then rinsed once again in PBS, for 5 minutes at room temperature. The slide were washed at 55°C in 2xSSC for 5 minutes, 1xSSC for 5 minutes, 0.5xSSC twice for 5 minutes and twice in 0.1× SSC/0.1% SDS for 5 minutes. The slides were rinsed in Wash Buffer 1 (100ml 10x maelic acid, 897ml MQ water, 3ml Triton X), and then incubated in 2% blocking solution (4g blocking reagent (Roche: # 11 096 176 001) in 200mls 1x maelic buffer) for 30 minutes at room temperature. Each tissue section was then incubated (already enclosed by Pap Pen) with 150µl alkaline phosphatase-labelled anti-DIG antibody (Roche: #11 093 274 910), at 1:500 in blocking buffer for 2 hours at room temperature or overnight at 4 ^o^C. (Colour reaction appeared with 30 minutes). To remove non-specificing binding, the slide were washed in Wash Buffer 1 three times over a total of 40 minutes, then in Wash Buffer 2 (Tris HCl 100 mM, NaCl 100 mM, MgCl_2_ 50 mM, pH 9.5) for 10 minutes. Each section was then covered with 200µl of freshly prepared colour solution (10ml Wash Buffer 2 with 200µl of a nitroblue tetrazolium chloride (NBT) / 5-bromo-4-chloro-3-indolyl-phosphate, 4-toluidine salt (BCIP) mix from Roche: #11 681 451 001), and 10µl 1M Levamisole (Sigma: # L9756) for final concentration of 1mM). Colour development appeared within 30 minute to an hour, when left at room temperature. The reaction was stopped by rinsing in Wash Buffer 3 (10mM Tris-HCl, pH 8.1, 1mM EDTA), followed by two 15 minutes washes in deionised water to remove Tris. Non-specific staining was further removed by using a series of three-minute ethanol washes (50%, 70%, 90%, 100%, 90%, 70% and 50%), and finally water. Slides were mounted with aqueous mounting medium (BDH Laboratory Supplies: #36226H) and sealed with glass cover slips. Images were captured using bright field illumination microscopy.
